# Infra-slow EEG neurofeedback for insomnia: a single-case experimental study in primary care

**DOI:** 10.3389/fnhum.2026.1832178

**Published:** 2026-07-16

**Authors:** Arne Edvardsson, Michael G. Smith, Sindre Engelbrekt Rolstad, Steinn Steingrimsson, Stefanie Enriquez-Geppert, Karlien Balt, Jonas Landahl, Sandra Weineland

**Affiliations:** 1 School of Public Health and Community Medicine, Institute of Medicine, Sahlgrenska Academy, University of Gothenburg, Gothenburg, Sweden; 2 Research and Development, Primary Health Care Region Västra Götaland, Borås, Sweden; 3 Department of Psychology, Faculty of Social Science, University of Gothenburg, Gothenburg, Sweden; 4 Department of Psychiatry for Affective Disorders, Region Västra Götaland, Sahlgrenska University Hospital, Gothenburg, Sweden; 5 Section of Psychiatry and Neurochemistry, Institute of Neuroscience and Physiology, Sahlgrenska Academy, University of Gothenburg, Gothenburg, Sweden; 6 Department of Clinical and Developmental Neuropsychology, Faculty of Behavioural and Social Sciences, University of Groningen, Groningen, Netherlands; 7 Department of Human Physiology, Faculty of Health Sciences, University of Pretoria, Pretoria, South Africa; 8 Center for Digital Health, Sahlgrenska University Hospital, Gothenburg, Region Västra Götaland, Sweden; 9 sensLab, Division of Digital Development and Innovation, Sahlgrenska University Hospital, Gothenburg, Region Västra Götaland, Sweden

**Keywords:** activity tracker, brain activity, complex interventions, EEG-biofeedback, infraslow oscillations, insomnia, primary care, sleep health

## Abstract

**Background:**

Insomnia is a prevalent and burdensome condition in primary care, associated with impaired functioning, increased health risks, and suffering. Infra-slow neurofeedback (ISF-NF) has been proposed as an intervention for stress-related sleep dysregulation, yet empirical evidence from primary care remains scarce.

**Objective:**

To examine the feasibility, acceptability, and preliminary effects of ISF-NF for insomnia in primary care using a single-case experimental design.

**Methods:**

A single-case experimental design with repeated measures was applied. Ten patients with insomnia and related comorbidities (e.g., stress-related exhaustion, migraine, anxiety) were enrolled. Nine completed the intervention, and eight had sufficient data for analysis following a 2-week baseline and 12 weekly ISF-NF sessions with individualized adjustment of the temporal integration (frequency) parameter. Subjective sleep quality was assessed daily using the Brief Self-Reported Sleep Quality Assessment (BSRSQA). Standardized questionnaires including the Pittsburgh Sleep Quality Index (PSQI), Patient Health Questionnaire-9 (PHQ-9), and Generalized Anxiety Disorder-7 (GAD-7) were collected at baseline, post-intervention, and 3-month follow-up. Treatment effects were evaluated using visual analysis and non-parametric Tau-U statistics. Wearable sleep data (Fitbit Inspire 3) were analyzed descriptively to contextualize subjective outcomes.

**Results:**

Visual single-case analyses revealed heterogeneous but interpretable trajectories, with several participants showing improved perceived sleep quality. Five of eight participants demonstrated statistically significant Tau-U effects (*p* < 0.05). Group-level analyses showed reductions in sleep problems (PSQI) and depressive symptoms (PHQ-9) from pre to post-intervention, with partial return toward baseline at three-month follow-up. Wearable sleep metrics indicated variability and limited convergence with total sleep time but suggested improvements in sleep consolidation and timing in some cases. Feasibility and acceptability were supported by high treatment adherence, therapeutic alliance, and few reported adverse effects.

**Conclusion:**

These findings suggest that ISF-NF may be a feasible and well-tolerated intervention in a primary care context, with preliminary indications of benefit in a subset of individuals. Effects were heterogeneous and should be interpreted cautiously. Individualized parameter adjustment and visual single-case analysis may provide a useful framework for capturing treatment dynamics in adaptive interventions. Wearable sleep data may offer contextual information but should be interpreted alongside subjective outcomes. Controlled studies are needed to evaluate efficacy and underlying mechanisms.

## 1 Introduction

Insomnia refers to difficulties with sleep quality or quantity, such as trouble falling asleep, frequent awakenings, or early morning awakenings with an inability to fall back asleep (American Psychiatric Association, 2013). When these issues occur at least three times per week for 3 months and interfere with daily functioning, the criteria for insomnia are met. The direct and indirect costs of insomnia in the United States are estimated to exceed $100 billion annually (Wickwire et al., 2016). Population-based surveys using standardized diagnostic criteria suggest that approximately 10–20% of adults meet case-level criteria for insomnia, with higher prevalence estimates reported when broader operational definitions are applied, such as questionnaire items mapped to ICSD-3 criteria (Zeng et al., 2020; Sivertsen et al., 2021; Porcheret et al., 2024). When insomnia disorder is more strictly defined to require both daytime impairment and adequate sleep opportunity, pooled prevalence estimates are lower, at approximately 12.4% in the general population (Van Straten et al., 2025).

Despite its high prevalence, insomnia remains substantially underdiagnosed and undertreated. Many affected individuals do not seek clinical care and may be unaware that their sleep difficulties meet diagnostic criteria for a psychiatric disorder (Bhaskar et al., 2016). Elevated rates of sleep problems have also been observed in specific populations, such as students and working professionals, where lifestyle-related factors including psychosocial stress and intensive digital technology use may further exacerbate sleep disruption. For example, Janatmakan Amiri et al. (2020) reported that approximately half of all respondents (medical students) had sleep problems, particularly those with higher mobile phone use.

Such findings have contributed to a shift away from viewing insomnia as a unitary nocturnal symptom toward conceptualizing sleep health as a multidimensional and contextually embedded construct. Recent work has formalized this perspective by defining sleep health as comprising multiple interrelated dimensions, including sleep duration, continuity, timing, regularity, subjective satisfaction, and daytime functioning (St-Onge et al., 2025). Within this biopsychosocial framework, sleep is understood as an emergent process shaped by interactions between biological regulation, psychological functioning, social demands, and environmental conditions.

From this perspective, insomnia is not solely characterized by insufficient sleep duration but may arise from disturbances across several dimensions of sleep, including arousal regulation, sleep continuity, circadian timing, and perceived restoration. These disturbances are influenced by factors operating at multiple levels, such as individual stress reactivity, emotional regulation, work-related demands, caregiving responsibilities, and broader societal influences including organizational norms and digital technology use.

Physiological hyperarousal and impaired autonomic regulation have been proposed as key mechanisms linking contextual influences to persistent insomnia (Moore, 2022). Chronic stress exposure and sustained cognitive or emotional activation may compromise the nervous system’s capacity to downregulate, thereby interfering with transitions into restorative sleep states.

In turn, insufficient or fragmented sleep has been associated with adverse mental health outcomes, including increased risk for depression and anxiety (Jansson-Fröjmark and Lindblom, 2008; Yasugaki et al., 2025), as well as with somatic morbidity and inflammatory dysregulation, with experimental studies demonstrating that even short-term sleep restriction may induce sustained alterations in hematological recovery and immune function (Lasselin et al., 2015; Benz et al., 2023).

At the population level, sleep disturbances have also been linked to occupational stress and burnout. Membrive-Jiménez et al. (2022) found associations between sleep problems and burnout syndrome among nurses, highlighting the role of psychosocial work environments in shaping sleep health. From a public health perspective, improving sleep has therefore been proposed as a cost-effective target for intervention, with potential benefits spanning mental health, physical health, and work functioning (Wickwire et al., 2016).

Cognitive behavioral therapy for insomnia (CBT-I) yields robust short-term improvements in sleep efficiency, shorter sleep-onset latency, and reduced wake after sleep onset, with minimal change in total sleep time (Van der Zweerde et al., 2020). However, pooled effects diminish between 3 and 12 months (pooled Hedges g for insomnia severity: 0.64 at 3 months, 0.40 at 6 months, 0.25 at 12 months). Not all patients respond or remit: meta-analytic and trial data indicate remission rates around 35–40% at post-treatment (with considerable variability across populations) and response rates of about 60% in some settings, leaving a substantial minority as non-responders (Wu et al., 2015; El-Solh et al., 2025; Van Straten et al., 2025). Dropout is also nontrivial; in a primary-care trial of nurse-guided internet-delivered CBT-I, 68% completed the program (≈32% dropout) (Van der Zweerde et al., 2020), and in the meta-analysis by Wu and colleagues, attrition varied across trials, with several studies judged at risk of attrition bias (Wu et al., 2015). In a veterans cohort with comorbid insomnia and obstructive sleep apnea receiving brief behavioral therapy for insomnia, 43% did not respond (defined as a ≥ 8-point reduction on the Insomnia Severity Index, ISI), with non-European descent and shorter baseline sleep time predicting non-response, supporting efforts to phenotype patients and tailor care (El-Solh et al., 2025). In summary, a substantial proportion of patients neither respond nor maintain improvements over the long term (Wu et al., 2015; El-Solh et al., 2025). Nevertheless, CBT-I provides more durable benefits than hypnotics at longer-term follow-up (Mitchell et al., 2012).

Benzodiazepines and so-called Z-drugs, a class of commonly prescribed hypnotic medications, are frequently used for sleep-related problems (Dimsdale et al., 2007; SBU, 2010). Experimental and clinical studies indicate that even low doses can significantly disrupt sleep architecture, including a 30–50% reduction in deep sleep stages (Dimsdale et al., 2007). Such alterations may contribute to a negative feedback loop in which patients increase medication use in response to persistently poor sleep quality. These medications are associated with the development of tolerance and diminishing efficacy with prolonged use (Holbrook et al., 2001), as well as a range of short and long-term health risks. While observational studies have linked hypnotic use to increased risks of accidents and mortality (Kripke, 2016), more recent large-scale register studies suggest that much of this risk may reflect underlying vulnerability and circumstances surrounding treatment initiation rather than medication effects per se (e.g., Osler and Jørgensen, 2022; Rozing et al., 2023). Taken together, these findings underscore the need for sustainable, non-pharmacological interventions to reduce reliance on long-term pharmacotherapy for sleep disorders.

Against this background, neurofeedback (NF) training has recently been proposed as one such approach, particularly as a potential alternative or complement for patients who do not sufficiently respond to cognitive behavioral therapy for insomnia (CBT-I). To date, studies using NF to reduce symptoms of insomnia are promising (Bekker et al., 2021; Lambert-Beaudet et al., 2021; Moore, 2022). NF is a technique that measures brain activity (e.g., using EEG, hemoencephalography, or functional near-infrared spectroscopy) and provides real-time feedback to enhance self-regulation (Sulzer et al., 2024). This process typically involves visual or auditory cues. For example, a video plays when the participant’s brain activity meets a target pattern and pauses when it deviates (Enriquez-Geppert et al., 2017). Current research on EEG-based NF suggests that it improves cognitive functioning in individuals with ADHD and may enhance sleep quality in individuals with insomnia (Arns et al., 2009; Schabus et al., 2017). Arns et al. (2014) propose that improved sleep may mediate the cognitive benefits observed in ADHD following NF. Furthermore, Arns and Kenemans (2014) report that NF training can improve the density of sleep spindles, which are associated with sleep consolidation and cognitive restoration.

NF constitutes an overarching category that encompasses a wide range of brain measurement techniques and methods, many of which differ substantially in their theoretical foundations and implementations (Enriquez-Geppert et al., 2017; Omejc et al., 2018; Garcia Pimenta et al., 2021; Bazzana et al., 2022). These methods vary across multiple dimensions, such as the number and placement of electrodes, targeted frequency bands, adjustment criteria, theoretical rationale, and the nature of neurophysiological changes induced during or following training.

A specific form of EEG-based NF that has gained increasing attention targets very slow EEG dynamics below approximately 0.5 Hz, often described in the literature as infra-slow oscillations (ISOs). Experimental work suggests that slow fluctuations in brain activity may relate to large-scale regulatory processes involving neural, autonomic, and neurovascular dynamics (Palva and Palva, 2012; Watson, 2018). NF protocols designed to operate within this slow temporal range have been explored in several clinical contexts, including stress-related conditions and sleep disturbances (Balt et al., 2020; Bekker et al., 2021; Bazzana et al., 2022). However, the physiological sources of such very slow EEG signals remain heterogeneous, and scalp recordings may reflect a mixture of cortical and peripheral physiological processes. In practical applications, these parameters reflect algorithmic processing of slow signal dynamics rather than direct training of discrete oscillatory frequencies.

NF protocols targeting slow EEG dynamics have been associated with improvements across several domains, including ADHD, trauma-related symptoms, and sleep disturbances (Bazzana et al., 2022). One such approach is infra-slow NF (ISF-NF**)**, which is based on the principle that training parameters are individualized through systematic adjustment guided by in-session responses and post-session feedback (Balt et al., 2020; Bekker et al., 2021). ISF-NF differs from other infra-slow-based approaches, such as infra-low frequency (ILF) NF, primarily in its methodological strategy. Whereas ILF protocols typically explore a broad range of frequency parameters, ISF-NF operates within a more constrained and systematically defined frequency parameter range, facilitating the identification of individualized training parameters. In this context, the term ‘frequency parameter’ refers to a temporal integration setting within the feedback algorithm and should not be interpreted as direct training of a discrete oscillatory frequency. ISF-NF can also be implemented using Low Resolution Electromagnetic Tomography (LORETA) with multiple active sites (Adhia et al., 2023; Mathew et al., 2022).

Although numerous studies on NF targeting infra-slow EEG dynamics have reported improvements in sleep following intervention (Legarda et al., 2011; Orakpo et al., 2021; Fleischman, 2022; Kirk and Dahl, 2022; Moore, 2022; Rauter et al., 2022; Sasu, 2022; Spreyermann, 2022), only one study to date has used sleep as its primary outcome (Bekker et al., 2021). Primary care is typically the first point of contact for insomnia, making it a critical setting for evaluating both effectiveness and implementation. To our knowledge, no prior study has examined ISF-NF in primary care or integrated daily subjective and physiological sleep assessments. Daily high-frequency measures increase ecological validity, reduce recall bias, and sensitively capture within-person change.

The present study aimed to evaluate the preliminary clinical effects of ISF-NF on subjective sleep quality and well-being, and to assess feasibility, acceptability, and participant satisfaction in a primary care setting using a SCED.

The following research questions were explored at both group and individual levels: (1) What are the effects of ISF-NF on subjective sleep disturbances, and how are these changes reflected in objective sleep measures? (2) What are the effects of ISF-NF on symptoms of depression and anxiety? (3) Is ISF-NF feasible and acceptable in a primary care setting?

## 2 Methods

### 2.1 Design

The present study was based on a single-case experimental design (SCED) with repeated measures (Krasny-Pacini and Evans, 2018). The study followed an AB design. The baseline phase (A) lasted at least 14 days and included daily sleep measurements. The intervention phase (B) started with all participants receiving the ISF-NF intervention, beginning with basic sleep hygiene information. ISF-NF training was conducted once a week for 12 sessions. The study is part of a larger research project in which interviews conducted for another sub-study were also carried out.

### 2.2 Inclusion criteria

All patients were diagnosed with insomnia, aged between 18 and 75 years, and motivated to adhere to the study protocol.

### 2.3 Exclusion criteria

Exclusion criteria were moderate to severe psychiatric issues such as depression, anxiety disorders, neuropsychiatric conditions, psychotic disorders, substance abuse, or personality disorders. Individuals who could not, speak, or understand Swedish were excluded, as were those receiving psychological or physiotherapeutic treatment deemed likely to influence study outcomes. Ongoing malignant disease was also an exclusion criterion, as were abnormal brain activity during the initial EEG screening. Individuals with regular night-shift work without the opportunity to sleep during work hours were excluded.

### 2.4 Participants

**Figure 1:**
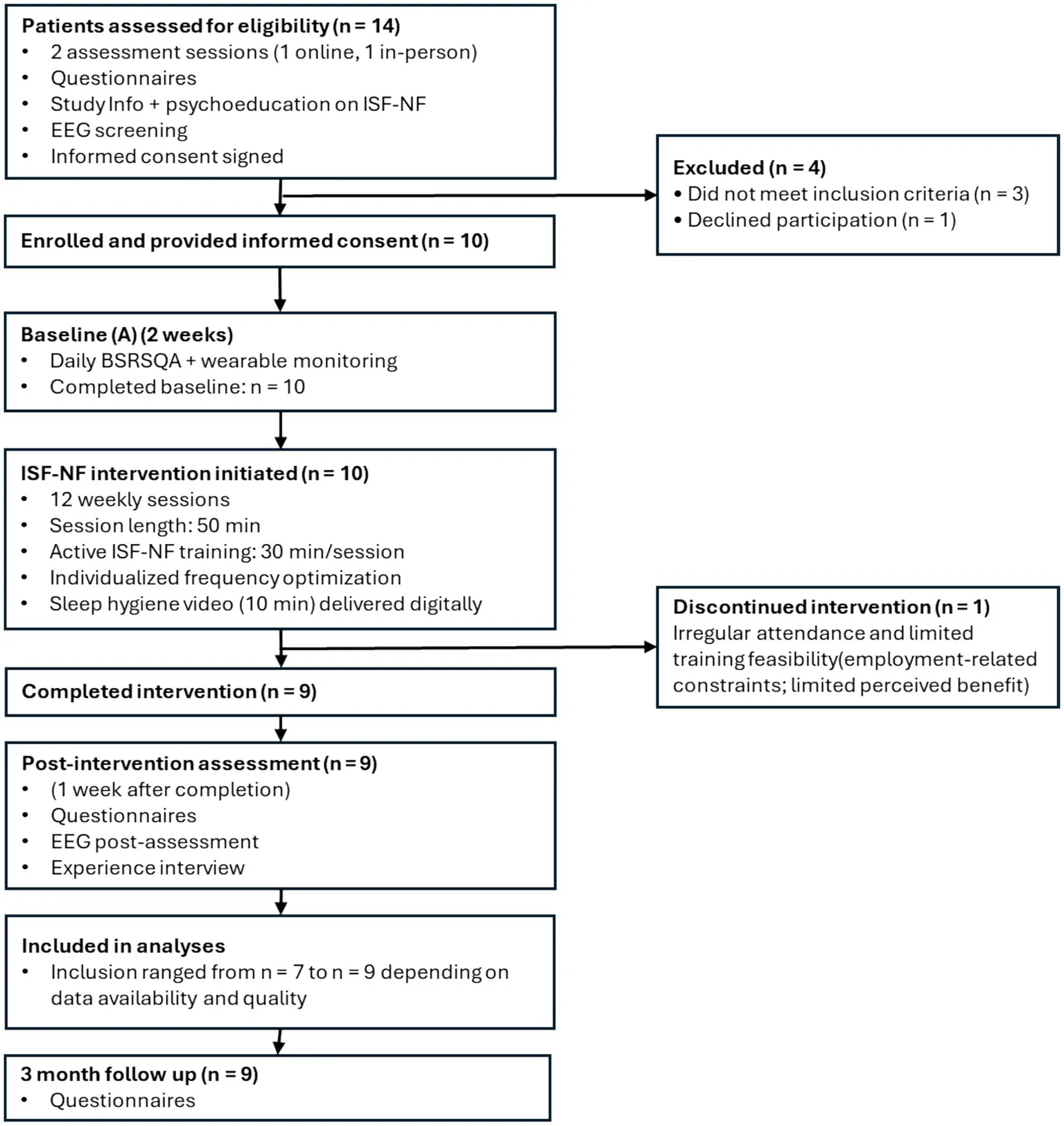
Study procedure, participant flow, and analysis inclusion. The figure illustrates participant assessment, enrollment, baseline, intervention, post-intervention, and follow-up phases, including intervention completion and attrition. Participant numbers (*n*) are shown for each phase. Inclusion in analyses varied across outcomes depending on data availability and quality, with sample sizes typically ranging from *n* = 7 to *n* = 9. The primary single-case experimental design (SCED) analyses were conducted on daily BSRSQA data. BSRSQA, Brief Self-Reported Sleep Quality Assessment; ISF-NF, infra-slow fluctuation neurofeedback; SCED, single-case experimental design.

Ten participants were recruited from the routine patient flow at a primary care center in Västra Götaland, Sweden, during 2024. In SCED research, evidence is typically established through systematic replication across a relatively small number of participants rather than through large sample sizes (Tate et al., 2017). The number of participants in the present study was therefore considered sufficient to allow replication of intervention effects across multiple individual time series. Participant characteristics are presented in Table 1, and the study procedure and participant flow are illustrated in Figure 1.

**Table 1:** Sample characteristics.

Variable	Description
Gender distribution	6 women, 4 men
Age	20–73 years (M = 48.4, SD = 16.64)
Insomnia duration	Varied from 4 months to 30 years: • < 1 year: 1 participant • ~ 2 years: 3 participants • ~ 5 years: 4 participants • > 10 years: 2 participants
History of insomnia treatment	5 participants
Medication use	• Any medication: 8 participants (e.g., Sertraline, Candesartan) • Sleep medication: 6 participants (e.g., Zopiclone)
Educational level	• Primary school: 1 • Secondary school: 6 • Higher education: 3
Occupational status	• Employed: 6 (including 3 on full-time sick leave) • Unemployed and job-seeking: 2 • Student: 1 • Retired:1
Comorbidities	All participants reported comorbidities including migraines (*n* = 4), pain (*n* = 3), menopausal symptoms (*n* = 5), anxiety (*n* = 5), burnout (*n* = 4). Participants also reported attentional and social communication difficulties, acute stress, and depressive and panic symptoms; when present, severity was generally mild.
Native language	All participants were native Swedish speakers.

Two participants had intermittent on-call night duties during the study period but did not meet criteria for regular shift work and were therefore retained in the sample. Sleep patterns for these participants were analyzed descriptively and interpreted in relation to work schedule variability. Participants were recruited through the routine patient flow at the primary care center.

### 2.5 Procedure

Participants were recruited during routine clinical care at a primary care center in Västra Götaland, Sweden, in 2024. Eligible patients were informed about the study by their physician, psychologist, or nurse at the health care center. Individuals who expressed interest were subsequently contacted by the project leader and received more detailed information about the study.

Data were collected using questionnaires and physiological measurements at multiple time points throughout the study (see Table 2).

**Table 2:** Data collection timeline across study phases.

Instrument/question	Pre-study	Baseline (A)	Intervention (B), daily	Intervention (B), post-session	Post-intervention	Follow-up (3 months)
DEM	x					
NF-SCL	x					
M. I. N. I.	x					
EXP-INT					x	
EEG	x				x	
ACT		x	x	x	x	
BSRSQA		x	x	x	x	
WAI-SR	x			x	x	
ISF-REP				x		
PSQI	x				x	x
PHQ-9	x				x	x
GAD-7	x				x	x
NEQ					x	
CSQ-8					x	

Questionnaires and study-related information were distributed via 1177.se, Sweden’s national publicly funded healthcare platform.

DEM, Demographics; NF-SCL, Neurofeedback symptom checklist; M. I. N. I, Mini international neuropsychiatric interview; EXP-INT, Semi-structured experience interview; EEG, electroencephalography screening (international 10–20 system: O1, O2, Cz, Fz); ACT, activity tracker–based sleep measurement; BSRSQA, brief self-reported sleep quality assessment; WAI-SR, working alliance inventory–short revised; ISF-REP, 24 h and 48 h post-session infra-slow fluctuation NF client report; PSQI, Pittsburgh sleep quality index; PHQ-9, patient health questionnaire–9; GAD-7, generalized anxiety disorder–7; NEQ, negative effects questionnaire; CSQ-8, client satisfaction questionnaire–8.

### 2.6 Intervention: ISF-NF training

#### 2.6.1 Equipment and software

The NF amplifier equipment used for EEG screening and ISF-NF training was a BrainMaster 2 EB + 2 × 2 two-channel EEG amplifier operated with BrainAvatar™ software (version 4.7.5; BrainMaster Technologies Inc., Bedford, OH, United States), together with the qEEG-Pro database developed in the Netherlands.

#### 2.6.2 Technical implementation of ISF-NF

NF training was delivered using auditory feedback generated through algorithmic processing of a DC-coupled bipolar EEG signal (T3–T4 montage). The bipolar montage reflects the difference in signal activity between the two recording sites, and feedback is based on how this differential signal evolves over time rather than on absolute activity at either site. The fast real-time signal represents the instantaneous magnitude of the amplitude difference between T3 and T4, increasing when the difference grows and decreasing when it diminishes. Feedback is generated by comparing this fast signal to a slower moving average, with changes in auditory output occurring when the fast signal crosses the slower reference curve (Balt et al., 2020; Bekker et al., 2021), where the slower reference signal is derived through exponential temporal integration across multiple time constants, typically in the range of approximately 5–20 s.

The feedback thus reflects directional changes and trends in the differential signal over time rather than its absolute amplitude, emphasizing temporal dynamics rather than static signal magnitude. In this context, the term “frequency parameter” refers to the temporal integration parameter within the feedback algorithm, defining the temporal scale of signal integration rather than a discrete oscillatory frequency. Accordingly, it should not be interpreted as training of a specific infra-slow rhythm.

The resulting feedback signal represents a composite slow EEG potential and may include contributions from both neural and non-neural physiological processes, such as slow cortical potentials, autonomic activity, neurovascular drift, and skin potential dynamics. No real-time separation between cortical infra-slow activity and peripheral physiological fluctuations was performed. Accordingly, the present protocol should be understood as a slow regulatory EEG feedback procedure rather than selective training of a discrete infra-slow oscillation. Although the precise physiological origins of this composite signal cannot be determined, the observed slow dynamics may nevertheless relate to underlying regulatory processes. Such regulatory dynamics may involve interacting neural and autonomic mechanisms, potentially including slow cortical potentials and processes such as the baroreflex. However, no specific physiological source can be isolated in the present setup.

Signal quality was continuously monitored throughout the sessions, both automatically by the software and manually by the therapist. The system flagged substantial signal contamination, including muscle activity and sweating, while the therapist monitored for additional artefacts such as eye movements or external disturbances.

#### 2.6.3 ISF-NF protocol

The ISF-NF training used in the present study was adapted from approaches previously implemented by Bekker et al. (2021) and Balt et al. (2020). If a participant missed a session, the intervention period was extended to allow completion of all 12 sessions.

For all participants, the initial placement of the active electrodes was T3–T4 according to the International 10–20 System. Sintered Ag/AgCl electrodes were used together with NeuroPrep scrub to ensure adequate adhesion to the scalp. Before each session, a signal test was conducted to verify that signal quality was suitable for training (impedance <10 kΩ).

During ISF-NF training sessions, participants were seated in a reclining chair while electrodes were positioned (three on the scalp: two active and one ground, and one electrode on each ear as reference). Either a headband or Ten20 conductive paste was used to ensure stable fixation of the electrodes. Participants were encouraged to make themselves comfortable and inform the therapist if they experienced any discomfort during the session, in which case immediate adjustments could be made. Training was conducted with eyes closed. Participants were instructed to simply sit quietly and listen to the auditory feedback signal. They were informed that the signal reflected ongoing brain activity and that they were not expected to actively control or manipulate it. Instead, participants were encouraged to adopt a relaxed and curious attitude while passively observing the feedback.

All participants began training using an initial temporal integration parameter corresponding to approximately 0.0062 Hz (or 0.0059 Hz in cases with a history of migraine), which has been reported to induce relaxation in most individuals (Karlien Balt, personal communication, June 1, 2023). This parameter represents a temporal integration setting within the feedback algorithm, defining the time scale over which slow fluctuations in the EEG signal are integrated for feedback generation.

During the course of training, the parameter was adjusted in small increments of 0.0001 Hz, either upward or downward, based on an iterative process combining therapist observation and participant-reported responses. During sessions, the therapist monitored observable indicators of autonomic state changes, such as breathing pattern, muscle tension, posture, and signs of increased relaxation or reduced arousal. Participants were also instructed to report any discomfort (e.g., tension, restlessness, nausea) during training.

In addition, participants completed structured symptom monitoring between sessions (Appendix E1), capturing indicators of both over and under-arousal. These reports were reviewed prior to subsequent sessions and informed parameter adjustments.

The aim of this iterative process was to identify an individualized parameter setting associated with a stable, tolerable, and subjectively beneficial state during training. This process was guided by clinical training and predefined symptom-monitoring criteria rather than fixed algorithmic rules.

To reduce the risk of circular interpretation, parameter adjustments were based on predefined symptom-monitoring domains (see Appendix E1) and observable in-session responses, rather than being determined directly from changes in the primary sleep outcome. These domains included indicators of both over and under-arousal, such as increased agitation, tension, or difficulty initiating sleep, as well as signs of sedation, nausea, gastrointestinal changes, or reduced perceived sleep depth. Parameter selection was further informed by ongoing clinical evaluation and discussion between the treating clinician and supervising expert, reflecting a structured combination of predefined criteria and clinical expertise.

#### 2.6.4 Therapist and supervision

The principal investigator responsible for assessment and intervention delivery (AE) was a licensed psychologist with more than 10 years of experience in NF. Supervision was provided by KB, co-developer of the ISF-NF protocol (see, e.g., Balt et al., 2020; Bekker et al., 2021).

### 2.7 Measurements

#### 2.7.1 Measurement instruments

Participants were asked to complete questions about housing, age, economic status, and medical history using a Demographic Questionnaire.

M. I. N. I. is a structured interview specifically developed to screen for DSM-IV and ICD-10 psychiatric diagnoses (Sheehan et al., 1998). Current versions are aligned with DSM-5 and retain an administration time of about 15 min (Lecrubier et al., 1997).

Daily subjective sleep quality was assessed with the BSRSQA, a brief four-item measure (1–7 per item) developed by the first and last authors for use in this study. As a study-developed instrument, it has not been previously validated. The measure was informed by existing subjective sleep assessments but was adapted for brevity and feasibility in daily repeated measurement. It was designed to capture within-person variation in perceived sleep quality over time, consistent with the single-case experimental design. Full item wording and scoring are provided in Appendix E3. The PSQI allows participants to evaluate their sleep quality over a one-month period (Buysse et al., 1989). It consists of 19 questions that can be divided into seven components. Buysse et al. (1989) reported that the PSQI had high sensitivity and specificity in distinguishing between good and poor sleepers.

The therapeutic alliance was measured after each intervention session using the WAI-SR (Munder et al., 2010). Responses are rated on a scale from 1 (not at all) to 7 (completely).

The PHQ-9 is an instrument used to screen for and assess the severity of depression based on DSM-IV criteria (Sun et al., 2020). Responses range from 0 to 3 (0 = not at all; 1 = several days; 2 = more than half the days; 3 = nearly every day). Participants are categorized into four levels of depression: 5–9 (mild), 10–14 (moderate), 15–19 (moderately severe), and 20 + (severe). Previous studies have shown a sensitivity of approximately 90% and a specificity of around 77–88% (Hansson et al., 2009).

The GAD-7 measures symptoms of anxiety (Spitzer et al., 2006). It includes seven standard questions and an optional eighth question about functional impairment. Participants respond to questions such as “Have you been feeling nervous, anxious, or on edge?” and “Have you had trouble relaxing?” on a scale from 0 (not at all) to 3 (nearly every day). The optional question asks, “If these symptoms occurred, how much did they affect your ability to work, carry out home tasks, and engage in relationships?” This is rated on a four-point scale from “not at all” to “extremely difficult.” The questionnaire has demonstrated strong psychometric properties (Johnson et al., 2019).

The CSQ-8 includes eight questions about patient satisfaction (Attkisson and Zwick, 1982). Responses range from 1 to 4, with 4 being the highest level of satisfaction. The questionnaire has shown high reliability and validity (Attkisson and Zwick, 1982).

The NEQ includes 32 questions about adverse events and effects of psychological treatment (Rozental et al., 2016). Participants rate the impact of negative treatment events on a scale from “not at all” to “extremely.” They are also asked whether they believe the negative effects were caused by the treatment.

### 2.8 Data collection

#### 2.8.1 Quantitative data collection using questionnaires

Questionnaires were administered through a survey service integrated into 1177, the Swedish national healthcare and information system.

#### 2.8.2 Activity tracker-based sleep data

Wearable-derived sleep data were collected using the Fitbit Inspire 3 to complement the primary subjective sleep outcomes with continuous, ecologically valid physiological measures of sleep. The device was used to capture total sleep time (TST), sleep timing, sleep consolidation, and fragmentation, and indices of nocturnal wakefulness, including wake after sleep onset (WASO) and the percentage of awake time during the sleep period (PADS), derived from stage-based data.

The Fitbit platform was selected because it is widely used in sleep and behavioral research, cost-effective, and provides raw data, enabling the development of a custom data extraction and monitoring solution in collaboration with sensLab at Sahlgrenska University Hospital. This facilitated continuous collection and preprocessing of wearable data across the full study period in a primary care context.

A previous model of this device family (Fitbit Inspire 2) has been validated against polysomnography (Lim et al., 2023), supporting its use for sleep monitoring in clinical research. The use of Fitbit Inspire 3 in the present study was therefore motivated by a combination of empirical support, technical accessibility, and feasibility for implementation in routine primary care. Current guidelines for the use of mobile health devices in intervention studies were followed (Balbim et al., 2021).

**Figure 2:**
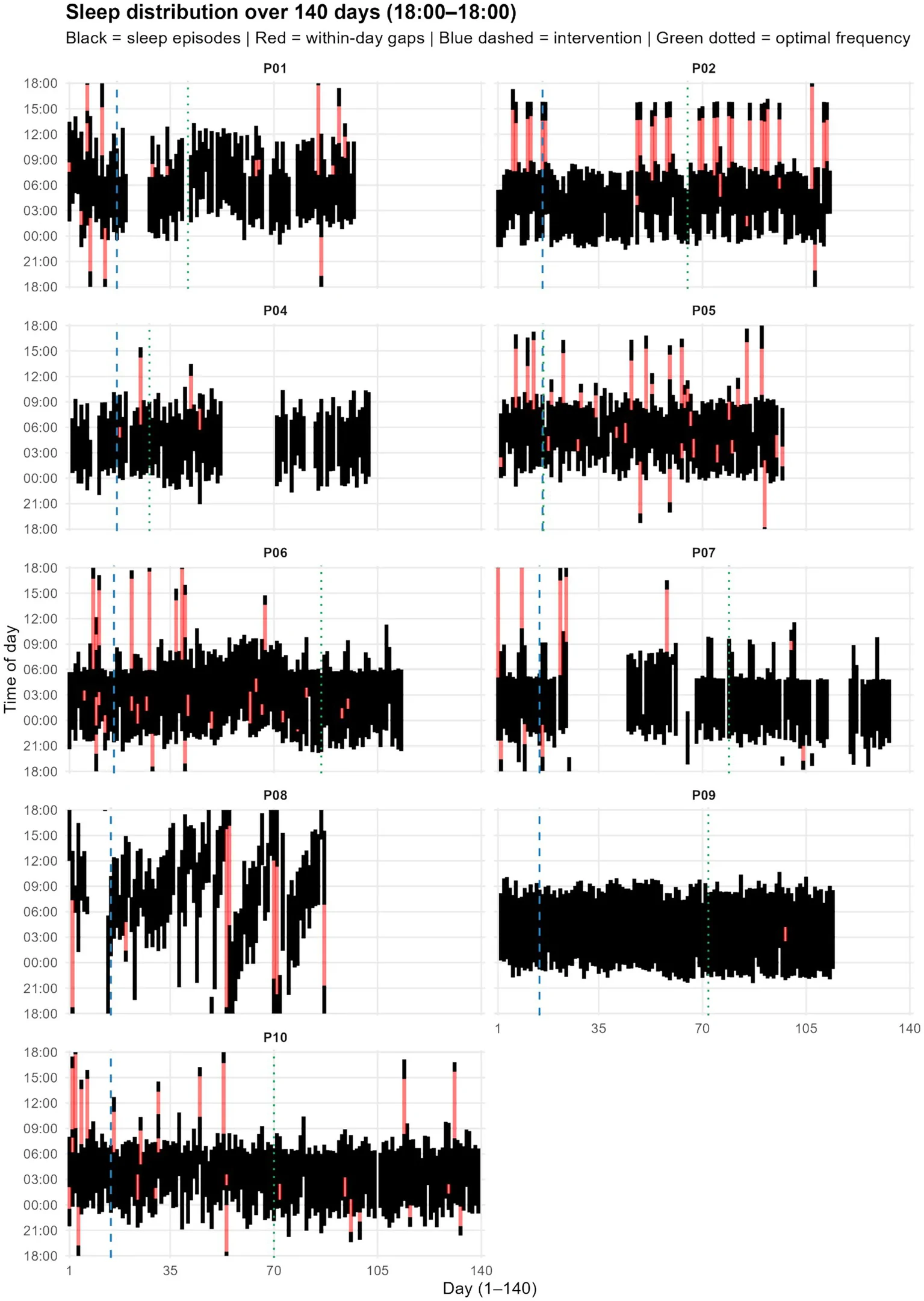
Sleep consolidation and timing plots across baseline and intervention phases. Actogram-style plots illustrate individual patterns of sleep timing and fragmentation across 140 days (18:00–18:00). Black bars represent sleep episodes derived from wearable data; red segments indicate within-day gaps. The blue dashed vertical line marks the onset of the intervention phase, and the green dotted vertical line indicates the time point at which an individualized optimal ISF-NF parameter setting was established. Individualized optimal parameter settings were reached at different time points across participants, representing participant-specific phase shifts. A descriptive participant-level summary is provided in Appendix C4.1.

Wearable-derived data were analyzed descriptively and at the single-case level to characterize individual patterns of sleep quantity, timing, and structure across baseline and intervention phases. An actogram-style visualization of sleep consolidation and timing patterns is presented in Figure 2, while additional objective sleep outcomes are reported in Appendix C2–C6. These analyses were intended to contextualize subjective sleep trajectories rather than evaluate group-level treatment efficacy. Variability in data completeness across participants due to temporary non-wear and technical factors is reported transparently in Appendix C1. All wearable-derived outcomes were treated as secondary, exploratory measures.

### 2.9 Data preparation

#### 2.9.1 Physiological and wearable recordings

EEG recordings were collected at baseline and post-intervention as part of the assessment protocol. These data are not analyzed in the present manuscript and are reserved for separate analyses focusing on neurophysiological mechanisms of ISF-NF. Accordingly, no direct conclusions regarding neural mechanisms can be drawn from the current results.

Wearable sleep recordings were obtained continuously during baseline and intervention phases using Fitbit Inspire 3 devices. These recordings were processed descriptively as specified in Appendix C and were used to contextualize subjective sleep outcomes rather than as primary endpoints.

#### 2.9.2 Questionnaire data handling

The Working Alliance Inventory—Short Revised (WAI-SR) includes two reverse-worded items (Items 4 and 10). Visual inspection identified isolated cases of likely misinterpretation, where a participant rated a reverse-worded item at the extreme low end while scoring all other items at the high end.

To address this, a conservative single-item imputation rule was applied. If a response to a reverse-worded item deviated by more than 1.5 standard deviations from the sample mean and by at least four scale points from the participant’s mean on the remaining WAI-SR items at the same time point, the value was flagged as a probable misinterpretation and replaced by the participant’s mean score across the remaining items (rounded to the nearest whole number). This approach preserves within-participant data while minimizing the impact of isolated response errors (Carlson et al., 2011).

### 2.10 Ethical considerations

The study was reviewed by the Swedish Ethical Review Authority (Etikprövningsnämnden) and approved with registration number (Dnr) 2023–05411-01. Following the initial online assessment, participants received additional study information and an informed consent form. Written informed consent was obtained during the subsequent in-person assessment before any intervention procedures began. After the study was completed, each participant was given the option to undergo a booster ISF-NF training session.

### 2.11 Data analysis

Analyses were conducted at the group level for questionnaire outcomes and at the individual level using SCED methods for daily BSRSQA ratings. Wearable-derived sleep data were analyzed descriptively as secondary outcomes to contextualize subjective sleep trajectories rather than evaluate treatment efficacy.

#### 2.11.1 Group-level questionnaire analyses

Group-level within-subject analyses were conducted on questionnaire outcomes assessing sleep problems, anxiety, and depression at baseline, post-intervention, and 3-month follow-up using a per-protocol approach. Non-parametric tests were applied due to the small sample size and non-normal distributions. For GAD-7 and PHQ-9, eight participants had complete data across all three time points and were included in the analyses. For PSQI, seven participants had complete data across all three time points and were included.

#### 2.11.2 SCED analyses of daily subjective sleep (BSRSQA)

##### 2.11.2.1 Visual analysis

Visual SCED analysis was conducted following the approaches described by Lane and Gast (2014) and Heyvaert and Onghena (2014). The independent variable was ISF-NF training, and the dependent variable was the daily BSRSQA sleep rating. Visual analysis was used to identify changes in the subjective sleep experience attributable to the intervention. For each participant, graphs displayed daily BSRSQA scores across all phases: baseline, the non-optimal parameter phase, and the phase with an individualized optimal ISF-NF parameter setting. ISF-NF sessions were denoted by vertical dashed lines. Each graph indicated the session at which the clinician established the participant’s individualized ISF-NF parameter setting based on participant reports and clinical judgment. Visual inspection focused on changes in level, trend, variability, and immediacy of effect across phases, with attention to delayed responses and potential deterioration patterns. This qualitative appraisal was complemented by quantitative metrics (see Section 2.11.2.2) to enhance interpretability. A standardized multi-panel figure illustrates all participants’ subjective sleep trajectories across the study period. Three cases were selected for detailed visual and narrative analysis with individualized axes and annotations. The remaining cases are reported in Appendix D3.

##### 2.11.2.2 Quantitative analysis (tau-U)

To complement the visual analysis, quantitative effect size estimation was conducted using Tau-U, a non-parametric statistic suitable for single-case experimental designs (Parker et al., 2011). Tau-U accounts for both non-overlap in data points between phases and trends within phases, offering a robust measure of change that adjusts for potential baseline trends.

Effect sizes were computed for each participant, comparing BSRSQA scores during the baseline phase to those in the intervention phase. Tau-U significance values were derived using one-tailed tests, and results were interpreted using conventional thresholds: small (≤ 0.20), moderate (0.21–0.59), and large (≥ 0.60). In parallel, standardized mean differences (Z-scores) were calculated to assess change in sleep quality, providing a complementary metric of effect size.

Given the SCED framework and the focus on within-person analyses, formal correction for multiple comparisons was not applied as part of the primary analysis. However, to increase methodological rigor and transparency, a supplementary Holm–Bonferroni correction was conducted. As this procedure is known to be conservative, it was used to evaluate the robustness of statistically significant findings rather than as a primary decision criterion.

#### 2.11.3 Wearable-derived objective sleep data (secondary, exploratory)

Wearable-derived sleep outcomes were included to provide an objective, continuous complement to the primary subjective sleep measures and to enhance ecological validity in a primary care context. Given the exploratory nature of these data and the known limitations of consumer-grade devices, all wearable analyses were interpreted descriptively and at the individual level, without inferential claims regarding treatment efficacy.

##### 2.11.3.1 Measures and operationalization

Wearable-derived sleep outcomes were analyzed as secondary, exploratory measures to characterize individual patterns of sleep quantity, continuity, consolidation, and timing across baseline and intervention phases.

Four complementary dimensions were derived from Fitbit sleep outputs:

1. Total sleep time (TST): total minutes classified as sleep per 24-h period, including nocturnal sleep and daytime sleep episodes.
2. Within-sleep wakefulness (PADS): percentage of awake-stage minutes within the sleep period.
3. Sleep consolidation: number of discrete sleep episodes per 24-h period, with fewer episodes interpreted as more consolidated sleep.
4. Sleep timing and regularity: temporal distribution of sleep episodes across the 24-h day, examined using actogram-style visualizations to assess regularity, phase shifts, and clustering across days.

##### 2.11.3.2 Visual analysis and actograms

Visual inspection of wearable-derived sleep time series was conducted as an integral part of the individual-level single-case analyses. For each participant, daily values of total sleep time (TST), total wake time after sleep onset (minutes Awake), and sleep fragmentation were plotted across baseline and intervention phases.

Visual analysis focused on within-person changes in level, variability, and temporal organization of sleep across days, including shifts in consolidation and regularity over the course of the intervention. Actogram-style visualizations were used to examine sleep timing and distribution across the 24-h day.

This visual appraisal served to identify clinically meaningful patterns, irregularities, and potential artifacts, and to contextualize quantitative phase comparisons. Particular attention was paid to convergence and divergence between objective sleep patterns and subjective sleep quality trajectories.

##### 2.11.3.3 Quantitative single-case analysis of wearable-derived sleep parameters

To characterize phase-related changes in wearable-derived sleep metrics, non-parametric single-case Tau analyses were conducted comparing baseline and intervention phases for total sleep time (TST).

Given the exploratory nature of the wearable outcomes and the known variability of consumer-grade sleep measures, Tau values were interpreted descriptively, with emphasis on direction and magnitude of change rather than statistical significance. No pooled estimates or group-level inferential analyses were performed for wearable-derived parameters, in line with their intended role as secondary, exploratory, and hypothesis-generating outcomes.

Eight participants provided sufficient daily self-ratings (BSRSQA) for single-case visual analysis. One participant discontinued the intervention after six sessions, and one completed the intervention but discontinued daily self-ratings after the first week. These two participants were excluded from the main SCED analysis but are included in the Supplementary material for transparency.

For the wearable data (Fitbit Inspire 3), some points were missing due to device non-use or technical issues that led to the exclusion of additional cases from certain analyses. The number of participants included in each specific analysis is therefore reported separately.

#### 2.11.4 Handling of missing data and analysis sets

Missing data occurred primarily due to incomplete questionnaire responses, temporary interruptions or non-wear of wearable devices, synchronization issues, or insufficient data quality for specific outcomes. As a result, the number of participants included varied across outcome measures and analyses. Sample sizes are reported explicitly for each outcome and summarized in the participant flow diagram and supplementary material.

Participants received automated reminders via the Swedish national healthcare platform 1177 when questionnaires were not completed on time and were reminded during sessions to synchronize their wearable devices if data uploads were missing. All analyses were conducted using available data without listwise deletion. Participants with partial data were retained in descriptive and supplementary analyses where appropriate.

#### 2.11.5 Data availability

The datasets generated and analysed during the current study are not publicly available. De-identified data relevant to the reported analyses may be made available by the corresponding author upon reasonable scientific request.

#### 2.11.6 CRED-nf considerations

The study has been designed and will be reported with reference to the CRED-nf recommendations (Ros et al., 2020), which provide guidance on transparency and methodological reporting in clinical NF research. Relevant checklist domains have informed the specification of outcome measures, feedback parameters, data processing procedures, and analytic strategies. An overview of how the CRED-nf items are addressed in the present study is provided in Appendix F1.

## 3 Results

### 3.1 Descriptive characteristics and treatment completion

#### 3.1.1 Sample overview

Nine of the ten participants completed the full NF intervention. Eight participants provided sufficient daily BSRSQA ratings to be included in the primary single-case experimental design (SCED) analyses. One participant discontinued the intervention after six sessions, and one participant discontinued daily ratings early in the study. Both participants were excluded from the primary SCED analyses but were retained in supplementary descriptive materials for transparency.

#### 3.1.2 Data availability and inclusion across analyses

Data availability varied across outcome domains. SCED analyses were conducted for participants with sufficient daily BSRSQA data, whereas group-level analyses were based on available pre and post-intervention questionnaire data.

Wearable-derived sleep measures (Fitbit Inspire 3) were available for nine participants; data from one participant were not included due to accidental deletion. Additional missing observations occurred due to device non-use and technical issues, resulting in further case-wise variability across specific wearable-derived outcomes.

To improve transparency, participant inclusion across analyses and assigned response profiles are summarized in Table 3. The “Main Text” column indicates which cases were selected for detailed presentation, based on data completeness and their ability to illustrate distinct response trajectories.

**Table 3:** Overview of participant inclusion across analyses and assigned response profiles.

**Participant**	**Completed**	**SCED (BSRSQA)**	**Group-level (PSQI/PHQ/GAD)**	**Main text**	**Response profile**	**Notes**
P01	Yes	Yes	Yes	Yes	Strong responder	Complete data
P02	Yes	Yes	Yes	No	Strong responder	Complete data
P03	Yes	Yes	Partial (missing post-intervention data)	No	No clear improvement	Missing post-intervention questionnaire data and wearable data
P04	Yes	No	Yes	No	Not classifiable (insufficient SCED data)	Limited BSRSQA data (insufficient for SCED analysis); partial Fitbit data
P05	Yes	Yes	Yes	No	Gradual responder	Complete data
P06	Yes	Yes	Yes	Yes	Strong responder	Complete data
P07	Yes	Yes	Partial (missing baseline data)	No	Strong responder	Missing baseline PSQI; comorbid sleep apnea identified post-intervention
P08	No	No	No	No	Not classifiable (early dropout)	Discontinued intervention; insufficient data for analysis; signal affected by sweat-related artifacts
P09	Yes	Yes	Yes	No	Strong responder	Complete data
P10	Yes	Yes	Yes	Yes	Non-sleep responder	Limited sleep improvement but meaningful clinical changes

Case selection for the main text was based on data completeness, intervention adherence, and the ability to illustrate distinct response patterns, with the aim of representing heterogeneous trajectories while maintaining readability of the main manuscript. Response profiles were informed by Tau-U estimates and visual SCED analysis, and should be interpreted descriptively. The SCED column indicates inclusion in single-case analyses based on daily BSRSQA data, whereas the group-level column reflects inclusion in pre–post questionnaire analyses. The “Main Text” column indicates cases selected for detailed presentation based on data completeness and their ability to illustrate distinct response patterns.

Detailed information on wearable-derived sleep data and data availability is provided in Appendix C, and participant-level completeness across measures is summarized in Appendix D1.

### 3.2 Changes in sleep, anxiety, and depression (group-level questionnaire outcomes)

#### 3.2.1 Descriptive statistics of questionnaire data

Descriptive statistics for all questionnaire-based outcomes are presented in Table 4.

**Table 4:** Descriptives for PSQI, GAD-7, PHQ-9, WAI-SR, CSQ-8, and NEQ.

Measure	Time point	n	M	SD	Median	Range
PSQI—sleep quality	Pre	9	13.89	3.76	14.00	08–19
	Post	8	09.00	4.07	8.50	04–15
	3-Month	09	11.00	4.61	10.00	04–17
GAD-7—anxiety	Pre	10	5.30	5.10	3.00	0–14
	Post	8	2.50	2.73	2.00	0–07
	3-Month	9	4.44	5.15	3.00	0–13
PHQ-9—depression	Pre	10	10.30	5.23	10.50	02–19
	Post	8	5.63	3.50	5.50	01–10
	3-Month	9	9.44	7.11	7.00	02–21
WAI-SR—working alliance	Mean across the intervention	10	70.82	10.51	72.73	44–84
CSQ-8—satisfaction	Post	8	25.50	3.93	23.50	21–32
NEQ—negative effects per participant	Post	8	1.75	1.91	1.50	0–5
NEQ—total perceived severity	Post	8	0.86	0.77	1.00	0–2

Measures included PSQI (0–21, higher = poorer sleep), GAD-7 (0–21, higher = more anxiety), PHQ-9 (0–27, higher = more depression), WAI-SR (12–84, higher = stronger alliance; per participant we averaged totals across 12 sessions to obtain an individual mean, then summarized these means at group level: M, SD, median, range), CSQ-8 (8–32, higher = greater satisfaction), NEQ number of effects (0–32) and NEQ severity (0–128, higher = more or more severe negative effects).

The eight individual responses that were corrected increased total WAI-SR scores by 4.88 points (range: 2–6 points), without affecting the overall pattern of alliance ratings over time.

#### 3.2.2 Sleep quality, anxiety, and depressive symptoms: group-level analysis based on a per-protocol approach

A Friedman test on PSQI scores revealed a statistically significant difference across the three time points, *χ*^2^(2, *n* = 7) = 10.296, *p* = 0.006. Post-hoc pairwise comparisons using Wilcoxon signed-rank tests showed significant reductions in PSQI scores between pre and posttest (*p* = 0.018), pretest and 3-month follow-up (*p* = 0.016), and between posttest and follow-up (*p* = 0.049). All comparisons remained statistically significant after Holm-Bonferroni correction.

Median PSQI decreased from baseline to post-treatment, but rose slightly at the 3-month follow-up, suggesting a partial loss of gains. Mean PSQI values remained above the conventional clinical cut-off of 5 (Buysse et al., 1989), indicating persisting clinically relevant sleep disturbance.

For PHQ-9, the Friedman test indicated a statistically significant difference across the three time points, *χ*^2^(2, *n* = 8) = 8.867, *p* = 0.012. Mean ranks were 2.56 for pretest, 1.19 for posttest, and 2.25 for the 3-month follow-up, suggesting a decrease in depressive symptoms during treatment, followed by a partial return of symptoms after the intervention ended.

Post-hoc Wilcoxon signed-rank tests showed a significant reduction in PHQ-9 scores from pre and posttest (*Z* = −2.524, *p* = 0.012), which remained significant after Holm-Bonferroni correction. No statistically significant differences were found between pretest and follow-up (*p* = 0.779), or between posttest and follow-up (*p* = 0.090).

The median PHQ-9 score at posttest was 5.50, with an interquartile range (IQR) of 2.25 to 9.50, indicating a substantial reduction in depressive symptoms. This suggests that the intervention may have contributed to a reduction symptom severity, with most participants scoring in the minimal to mild depression range post-intervention.

At the 3-month follow-up, there was a partial relapse in depressive symptoms, as scores increased compared to the post-intervention assessment but remained lower than baseline levels. The median score at follow-up was 8.00, with an IQR of 2.50 to 17.75. The large standard deviation and wide interquartile range at follow-up suggest increased variability in symptom trajectories among participants.

The descriptive statistics for GAD-7 scores before the intervention, after the intervention, and at the three-month follow-up indicate notable changes in anxiety symptoms over time (Table 5). Before the intervention, participants exhibited mild to moderate anxiety symptoms, with considerable inter-individual variability. Following the intervention, the mean GAD-7 score decreased, and the median score dropped to 2.00 (IQR = 0.00–5.25), indicating a substantial reduction in anxiety symptoms. This suggests that the intervention may have been associated with a short-term reduction in anxiety symptoms, with most participants falling within the minimal symptom range post-intervention.

**Table 5:** Quantitative single-case experimental design analysis of the brief self-reported sleep quality assessment for eight participants who completed the intervention and provided complete daily ratings.

Participant	Tau-U	Z	*p*-value	CI 90%
P01	0.6704	3.48	< 0.001***	0.354–0.987
P02	0.7684	4.78	< 0.001***	0.504–1.000
P03	0.0762	0.48	0.631	−0.185 – 0.337
P05	0.2921	1.80	0.073	0.025–0.560
P06	0.6026	3.76	< 0.001***	0.339–0.866
P07	0.6328	3.87	< 0.001***	0.364–0.902
P09	0.588	3.55	< 0.001***	0.316–0.860
P10	−0.1531	−0.94	0.348	−0.422 – 0.115

Asterisks indicate levels of statistical significance (* *p* < 0.05, ** *p* < 0.01, *** *p* < 0.001). Given the SCED framework and the focus on within-person analyses, formal correction for multiple comparisons was not applied as part of the primary analysis. However, to enhance methodological rigor, a supplementary Holm–Bonferroni correction was conducted to assess the robustness of statistically significant findings. All effects marked with *** remained significant after correction.

At the three-month follow-up, there was a slight increase in anxiety symptoms, suggesting a partial return of difficulties. The median score was 3.00, with an interquartile range of 0.75 to 11.00. The increased standard deviation and IQR at follow-up reflect greater variability in symptom trajectories, with some participants maintaining improvements and others experiencing a resurgence of anxiety.

However, the Friedman test indicated no statistically significant differences in anxiety symptoms across the three time points, *χ^2^*(2, *n* = 8) = 4.357, *p* = 0.113. Mean ranks were 2.31 for pretest, 1.44 for posttest, and 2.25 for follow-up, indicating a descriptive trend of reduced anxiety following the intervention, which partially reversed at follow-up. Given the lack of overall statistical significance, no post-hoc pairwise comparisons were conducted.

#### 3.2.3 Treatment engagement, Alliance, satisfaction and negative effects

Out of the ten participants who consented for treatment, 9 went through the whole treatment (90% adherence). Participant satisfaction was high on the CSQ-8 (*n* = 8; mean item score 3.19/4; Appendix B3). All participants (8/8) rated service quality as good or excellent and would recommend the service; 7/8 reported that their needs were met (definitely or generally), and 8/8 were very or mostly satisfied with the amount of help received. In general, there were few experiences of negative effects reported by the participants’ after participating and no rated no severe effect from the ISF-NF training, measured by the NEQ (see Appendix B2).

While the questionnaire outcomes provide a group-level summary of clinical change, they do not capture the temporal dynamics of sleep improvement. To address this, individual treatment trajectories were examined using single-case experimental design analyses of daily subjective sleep ratings.

### 3.3 SCED results: individual sleep trajectories

#### 3.3.1 Overview of individual trajectories in subjective sleep quality (BSRSQA)

**Figure 3:**
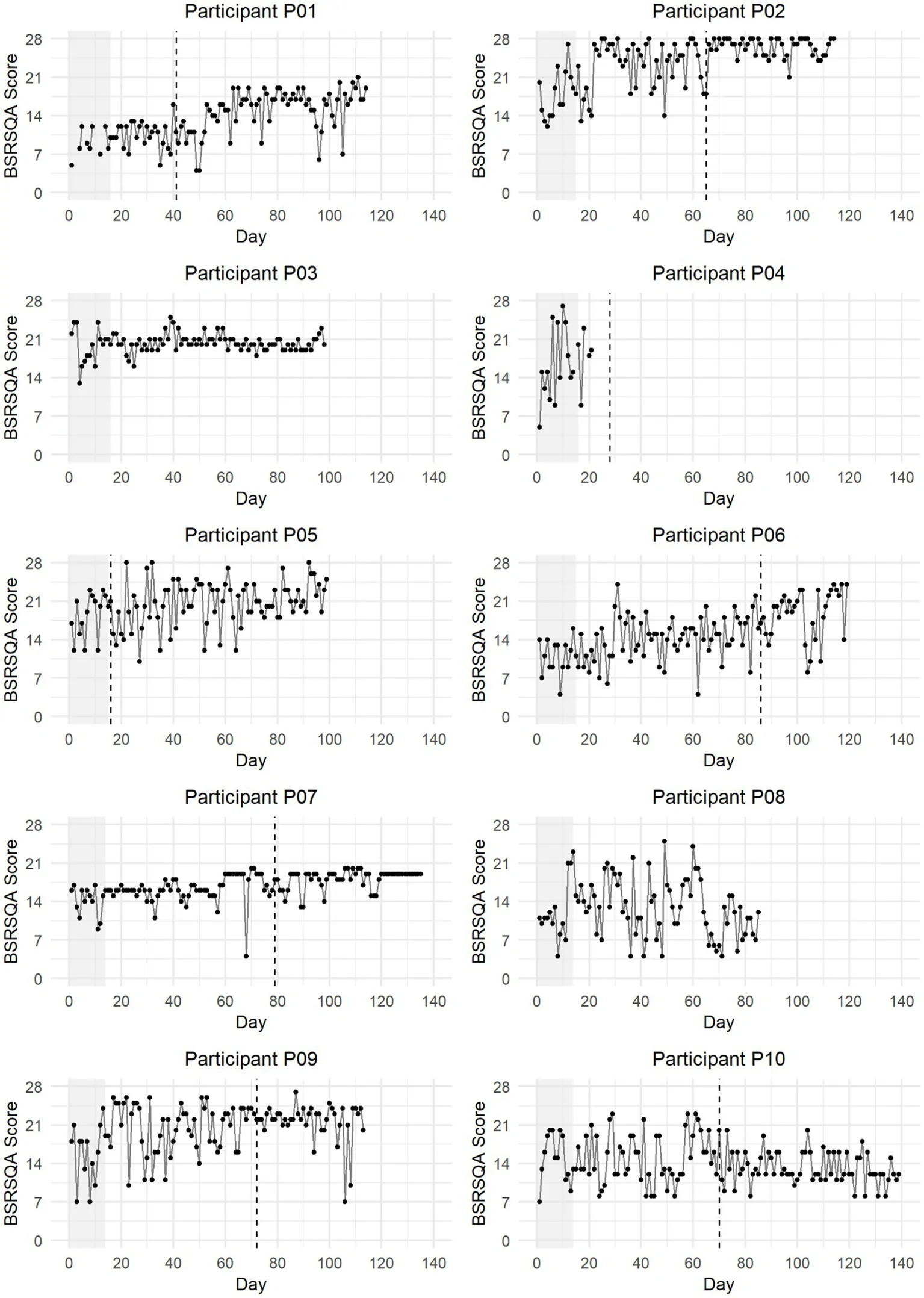
Daily BSRSQA scores (subjective sleep quality) for all participants (P01–P10) across the study period. The gray shaded area represents the baseline phase, and the white area represents the intervention phase. Each point corresponds to one daily BSRSQA self-rating (range: 0–28), with higher scores indicating better perceived sleep quality. Lines connect consecutive observations to facilitate visual inspection of within-participant trends over time. Vertical dashed lines indicate the time point at which an individualized optimal ISF-NF parameter setting was identified for each participant, where applicable. Participant P04 continued the intervention but discontinued completion of daily BSRSQA ratings during the later phase of the study. Participant P08 discontinued participation before completing the full intervention period.

In general, the individual graphs for BSRSQA indicate that most participants improved their perceived sleep (see Figure 3).

#### 3.3.2 Visual single-case analysis of BSRSQA time series

**Figure 4:**
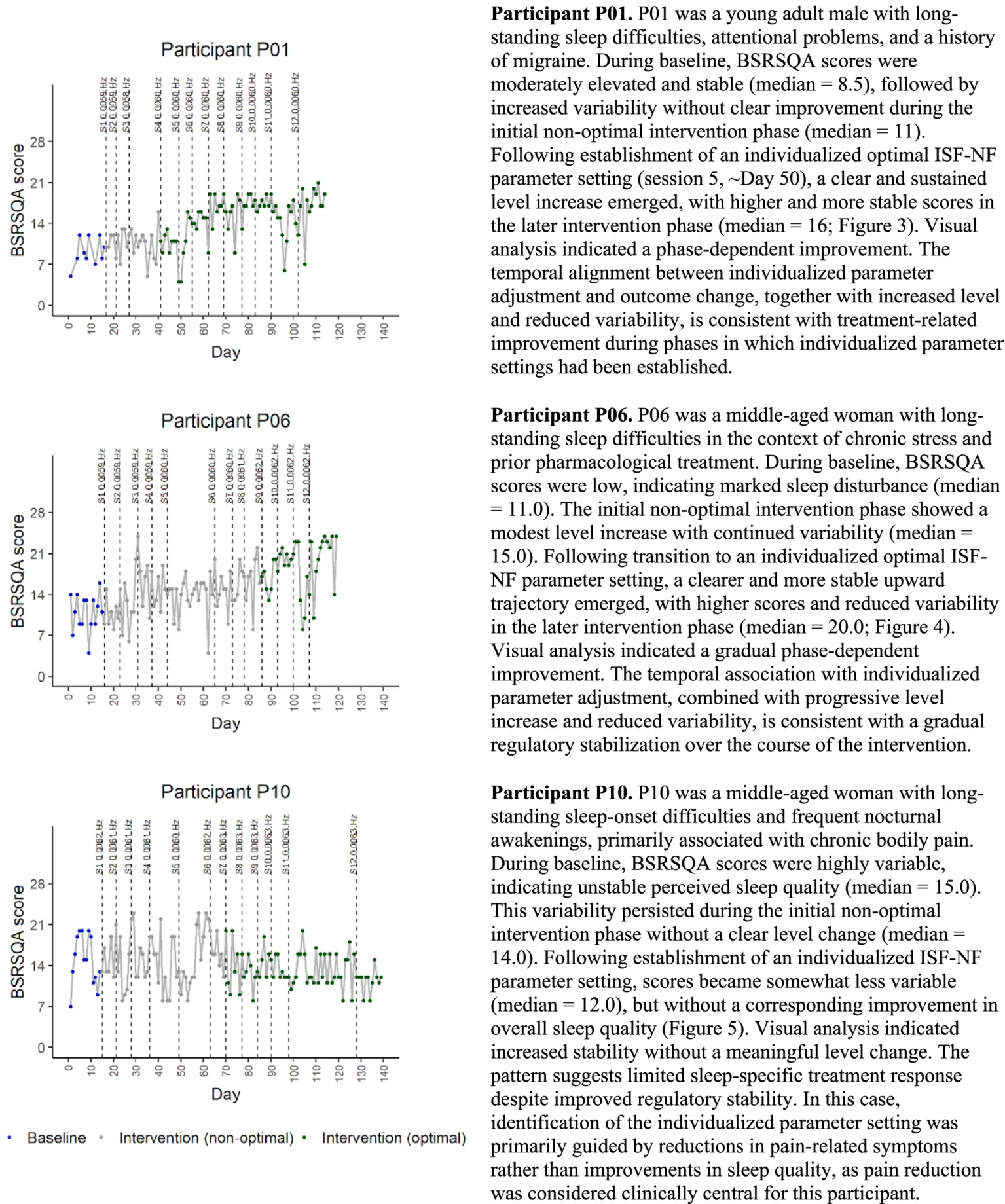
Daily BSRSQA scores for Participants P01, P06, and P10. Dotted vertical lines indicate ISF-NF training sessions and the corresponding temporal integration (frequency) parameter used during each session.

To illustrate individual response patterns to ISF-NF training, three participants (P01, P06, and P10) were purposefully selected for detailed visual and narrative presentation (see Figure 4). Selection was based on predefined criteria, including data completeness, intervention adherence, and representativeness of distinct response patterns (clear improvement, gradual improvement, and limited response), rather than random sampling. The selected cases did not differ meaningfully from the remaining participants with respect to key demographic characteristics or baseline clinical measures. A detailed rationale for inclusion in the main text versus appendix is provided in Appendix D2.

For participants not included in the main text, individual BSRSQA time series and concise visual single-case interpretations are presented in Appendix D3 (Visual BSRSQA analysis—Participants Not Included in Main Text). This appendix includes brief case summaries, corresponding figures (Supplementary Figures D3a–D3g), and a structured overview of baseline patterns, phase-related changes, and post-adjustment trajectories to support transparent comparison across cases.

For each selected participant, visual analysis of daily BSRSQA time series was conducted in accordance with established single-case design principles, focusing on baseline stability, level and variability during the non-optimal parameter phase, and changes temporally associated with the establishment of an individualized ISF-NF parameter setting. Median BSRSQA scores by phase and participant are reported in Appendix A1. Visual analysis served as the primary basis for single-case inference in the present section.

Each case vignette includes a brief participant description and a visual summary of BSRSQA trajectories. Questionnaire outcomes (PSQI, PHQ-9, and GAD-7) across timepoints are reported separately in Appendix B1, and diagnostic instruments are described in Appendix B. To maintain analytical clarity and methodological focus, qualitative interview findings are not included in the present article and will be reported in a separate qualitative publication addressing experiential and process-related aspects of ISF-NF training.

#### 3.3.3 Quantitative SCED analysis of BSRSQA time series

Eight participants who completed the intervention and provided daily BSRSQA ratings throughout the entire study period were included in the analyses (see Table 5). Five participants (P01, P02, P06, P07, and P09) demonstrated statistically significant improvements in perceived sleep quality (*p* < 0.05). One participant (P05) showed a positive trend that approached significance (*p* = 0.0725). Two participants (P03 and P10) did not exhibit statistically significant changes.

All Tau-U values and confidence intervals were computed using the non-parametric Tau-U calculator provided by the Single Case Research Center^1^. As per standard procedure, confidence intervals that mathematically exceeded the theoretical upper limit of Tau-U (1.0) were truncated at 1.0, as Tau-U values are bounded between −1.0 and 1.0.

In line with conventions implemented in this tool and commonly applied in single-case research, individual-level Tau-U estimates are reported with 90% confidence intervals. This level was chosen to enhance sensitivity and interpretability in small-N, within-person analyses, where the primary aim is to characterize individual change patterns rather than to draw population-level inferences.

The mean combined Tau-U value across participants was 0.43 (Z = 7.31, *p* < 0.001), indicating a moderate to strong effect size and suggesting potential clinical relevance of ISF-NF for insomnia in primary care. Participant P08 was excluded due to poor adherence, irregular attendance, and low training quality, while P04 lacked sufficient data during the intervention phase.

The Tau-U analysis indicated that a majority of participants experienced improvements in subjective sleep quality during the intervention phase, with effect sizes ranging from small to large. While the SCED analyses focused on perceived sleep quality as the primary outcome, wearable-derived sleep data were included to complement these findings by providing information on sleep structure and timing at the individual level.

### 3.4 Secondary outcomes: wearable-derived objective sleep parameters (exploratory)

Wearable-derived sleep data were included as secondary, exploratory outcomes to complement the primary subjective single-case analyses. Given the single-case design, small sample size, and pronounced day-to-day variability in sleep parameters, these data were analyzed descriptively at the individual level. Data availability and completeness across participants are summarized in Appendix C.

#### 3.4.1 Total sleep time (TST)

Changes in total sleep time (TST) were heterogeneous, with no statistically significant effects observed across participants. Confidence intervals were wide, reflecting substantial day-to-day variability (Appendix C3.1).

#### 3.4.2 Sleep consolidation and timing

Visual inspection of actogram-style sleep timing plots revealed marked inter-individual differences in baseline sleep organization. Among participants who initially exhibited fragmented sleep characterized by multiple discrete nocturnal segments, a descriptive reduction in the number of separate sleep episodes was observed over the course of the intervention (see Figure 2), suggesting increased sleep consolidation in a subset of cases.

Importantly, this shift was not universal, and patterns varied depending on baseline sleep structure and contextual factors. Consolidation effects were most apparent in individuals with pronounced baseline fragmentation, whereas participants with already consolidated sleep showed minimal structural change. Additional participant-level actograms are provided in Appendix C4.1.

Exploratory analyses of nocturnal wakefulness (PADS) showed substantial variability and are interpreted cautiously given known limitations in wearable-derived staging accuracy (see Appendix C5.2).

#### 3.4.3 Integration of subjective and objective sleep outcomes

Integrated analyses indicated partial convergence between subjective and objective sleep outcomes. Improvements in perceived sleep quality were in several cases accompanied by improvements in sleep consolidation and/or timing, even in the absence of increased TST (see Appendix C6).

## 4 Discussion

### 4.1 Summary of main findings

This exploratory SCED study in primary care suggests that ISF-NF may be associated with improvements in perceived sleep quality in several participants. Visual inspection of daily BSRSQA trajectories, supported by Tau-U effects in several cases, revealed heterogeneous but interpretable within-person change. Secondary, exploratory activity-tracker–derived sleep data suggested recurring patterns of improved sleep consolidation and timing, whereas changes in total sleep time were inconsistent and generally non-significant. Objective and subjective sleep trajectories were not uniformly aligned and varied across cases.

At the group level, questionnaire data showed reductions in sleep problems (PSQI) and depressive symptoms (PHQ-9) from pre to post-intervention. However, these improvements showed partial return toward baseline levels at three-month follow-up. Anxiety symptoms (GAD-7) did not change significantly, consistent with relatively low baseline levels in several participants. Given the absence of a control group, these findings should be interpreted with caution.

Regarding feasibility and acceptability, WAI-SR scores were consistently high, satisfaction ratings derived from the CSQ-8 indicated positive treatment experiences, and the NEQ showed few reported adverse events. These findings suggest that ISF-NF may be a feasible and well-tolerated intervention in a primary care setting. These aspects of feasibility and acceptability should be interpreted separately from the preliminary indications of possible clinical benefit described above.

### 4.2 Individualized single-case analysis, parameter optimization, and heterogeneous trajectories

The individualized SCED framework allowed detailed examination of temporal change patterns within participants, providing insight into how treatment dynamics unfolded over time. Given the adaptive nature of ISF-NF and marked inter-individual differences in baseline regulation and response dynamics, analytic approaches that preserve within-person trajectories are particularly well suited to this research question (Molenaar and Campbell, 2009; Fisher et al., 2018). Visual analysis enabled examination of level, trend, variability, and phase-related shifts across the intervention, capturing clinically meaningful change patterns that would likely be obscured in aggregated or simple pre–post analyses.

Participants reached their individualized optimal ISF-NF parameter setting at different time points during the intervention, resulting in staggered phase shifts within individual trajectories. In several cases, visual inspection suggested that patterns of change became more stable and less fluctuating following establishment of this individualized optimal parameter setting. Although these observations do not permit definitive causal inference, they suggest that distinguishing total training exposure from exposure delivered under individualized optimal parameter settings may be relevant when interpreting adaptive ISF-NF protocols. Early intervention phases were sometimes characterized by instability or minimal change, followed by more consistent patterns during later phases once individualized optimal parameter settings had been established.

Heterogeneity was also evident in the longer-term course of sleep-related and associated mental health outcomes. During the intervention, reductions in perceived sleep difficulties were in several cases accompanied by parallel reductions in depressive symptoms. At three-month follow-up, partial return of sleep-related difficulties was observed in some individuals, in certain cases alongside increased depressive symptom burden. These patterns are consistent with longitudinal evidence supporting bidirectional associations between sleep disturbance and affective symptoms (Jansson-Fröjmark and Lindblom, 2008) and may reflect dynamic interactions between ongoing psychosocial stressors and sleep regulation rather than simple loss of treatment effect.

Taken together, these findings suggest that patterns of change following ISF-NF may follow multiple trajectories, shaped by the timing and duration of exposure to individualized optimal parameter settings as well as by broader contextual factors. Within the exploratory SCED framework, these observations indicate that both total training exposure and the timing of individualized parameter adjustment may be relevant for understanding treatment dynamics. Variation observed at follow-up may additionally reflect broader contextual factors that were not systematically assessed in the present study. Accordingly, these findings should be interpreted as preliminary and hypothesis-generating rather than as evidence of specific treatment effects.

### 4.3 Clinical relevance and multidimensional pathways of sleep improvement

Subjective sleep quality was the primary outcome of this study and remains central to the clinical definition of insomnia. Contemporary sleep health models conceptualize sleep as a multidimensional construct, encompassing interacting dimensions such as duration, continuity, timing, regularity, subjective satisfaction, and daytime functioning (St-Onge et al., 2025). Within this framework, subjective sleep quality constitutes a core dimension of sleep health, positioned alongside these other components. At the same time, it reflects the individual’s integrated appraisal of how these dimensions co-occur and interact over time, rather than any single parameter in isolation.

Improvements in subjective sleep quality were not uniformly mirrored by objective wearable-derived metrics. Changes in total sleep time were inconsistent across participants. However, a descriptive pattern emerged in several cases indicating improved sleep consolidation over the course of the intervention. Specifically, participants who initially exhibited multiple fragmented sleep segments per night tended to show fewer nocturnal sleep segments over time.

This shift toward fewer nocturnal segments suggests that changes in sleep organization, rather than total duration, may reflect one clinically relevant dimension of change. These patterns were not universal and should be interpreted descriptively within the exploratory scope of the study. The partial convergence and divergence between subjective and objective indices remains consistent with prior insomnia research demonstrating that perceived sleep quality does not map linearly onto single objective sleep parameters (Perlis et al., 2025).

### 4.4 Feasibility, acceptability, and therapeutic alliance

As reported above, satisfaction and alliance ratings were consistently high, and few adverse effects were observed. These findings represent an important aspect of feasibility and acceptability and should be interpreted independently of treatment effects. They may also be clinically relevant given the individualized and adaptive nature of ISF-NF (Bazzana et al., 2022). In ISF-NF practice, transient increases in instability or fluctuations in arousal may occur, particularly during early phases of ISF-NF frequency parameter adjustments (Balt et al., 2020; Bekker et al., 2021). Such responses are typically interpreted as signals guiding protocol modification rather than adverse effects per se.

The method involves ongoing collaboration between clinician and patient to identify and refine individualized training parameters, including the establishment of an individualized optimal ISF-NF parameter setting. This adaptive process places particular emphasis on clinician competence and relational stability. Several participants reported increased emotional receptivity following sessions, including grief and heightened bodily awareness. These responses sometimes co-occurred with subjective sleep improvements and at other times occurred independently of changes in sleep quality.

Sessions involve gradual downregulation of arousal and shifts toward states approaching sleep onset, often accompanied by increased interoceptive sensitivity. Entering such states may occur more readily within a context characterized by safety and trust. Together, these observations underscore the importance of structured training, supervision, and careful monitoring when implementing ISF-NF in healthcare settings.

### 4.5 Neurophysiological context of infra-slow NF effects

In the present protocol, the feedback signal represents a composite slow EEG potential derived from DC-coupled bipolar recordings and may include contributions from both neural and non-neural physiological processes. Such slow signals can reflect a mixture of slow cortical potentials, autonomic activity, neurovascular drift, and skin potential dynamics. No real-time separation between cortical infra-slow activity and peripheral physiological fluctuations was performed.

This complexity is further illustrated by a practical observation in one participant who discontinued the intervention early, where excessive sweating repeatedly compromised signal stability and produced large slow fluctuations in the EEG signal, limiting training quality. This example highlights how very slow EEG recordings may be influenced by peripheral physiological factors.

Within this context, the gradual and individualized response trajectories observed in the present study may reflect changes in slow regulatory dynamics rather than modulation of a single, specific oscillatory process. Prior experimental and clinical studies of infra-low or infra-slow NF have reported changes in functional connectivity and autonomic physiology (Balt et al., 2020; Dobrushina et al., 2020, 2022; Bekker et al., 2021). While such findings provide a potential framework for interpreting behavioral and clinical changes observed following NF training, the proposed neurophysiological mechanisms in the present study remain hypothetical, as no concurrent neurophysiological measures were included to directly test these mechanisms.

Although EEG recordings were obtained before and after the intervention, these data were not analyzed as part of the present study and are reserved for separate analyses focusing on potential neurophysiological mechanisms. In addition, the present study did not examine whether the feedback variable itself showed systematic modulation within or across sessions. Consequently, it cannot be determined whether participants learned within the present dataset to regulate the EEG-derived feedback signal during training. Future work could examine such recordings to explore whether neurophysiological changes accompany clinical improvement. In particular, future studies should investigate NF learning dynamics systematically within and across sessions and examine whether changes in EEG-derived metrics parallel improvements in subjective sleep outcomes.

At a broader theoretical level, the gradual and parameter-dependent response patterns observed in the present study are compatible with existing models describing very slow fluctuations in brain activity and large-scale network dynamics (Palva and Palva, 2012; Watson, 2018). However, these theoretical frameworks should be considered interpretative context rather than direct evidence of the mechanisms underlying the present findings, and should therefore be understood as hypothesis-generating rather than explanatory.

### 4.6 Methodological considerations and limitations

The SCED framework allows fine-grained analysis of temporal patterns within individuals; however, replication in larger controlled studies is needed to strengthen external validity and confirm causal interpretations. The present study employed an AB single-case design with a baseline phase but without a controlled comparison condition, which limits causal inference and increases susceptibility to time-related and nonspecific effects. A multiple-baseline SCED design with staggered intervention onset across participants would have strengthened internal validity by introducing an element of experimental control and randomization. Baseline phases were relatively brief (2 weeks), which may reduce the stability of pre-intervention estimates, although no systematic pre-intervention trends were observed.

Furthermore, the sample consisted of participants who were motivated to engage in a relatively complex and time-intensive intervention. This may limit generalizability, as individuals who are more sceptical of NF or have lower adherence capacity may respond differently.

It should also be noted that the primary repeated outcome (BSRSQA) was a study-developed instrument without prior validation. While it was designed to capture within-person variation in perceived sleep quality in a high-frequency measurement context, this limits comparability with established measures and warrants cautious interpretation of the findings.

The integration of high-frequency sleep measurements with wearable-derived metrics represents a methodological strength, yet also delineates important boundary conditions. Consumer-grade devices such as the Fitbit Inspire series demonstrate acceptable validity for estimating total sleep time in healthy populations (Lim et al., 2023), but their performance is reduced in insomnia samples, where nocturnal wakefulness and sleep–wake misperception challenge algorithmic detection. Wearable-derived staging indices and composite metrics should therefore be interpreted as exploratory and descriptive rather than definitive markers of sleep physiology. Data completeness and quality also varied due to practical factors related to device use.

The intervention was delivered in a real-world primary care context, during which vacations and life events influenced sleep trajectories. While this enhances ecological validity, it complicates attribution of change to the intervention alone. Moreover, the individualized and therapist-guided nature of the intervention may further increase susceptibility to nonspecific influences. As in most individualized behavioral interventions, clinician-guided parameter adjustment may introduce additional nonspecific influences such as expectancy, therapeutic alliance, or increased self-monitoring. These factors may contribute to perceived improvements independently of the specific NF process of the feedback signal and were not directly controlled for in the present design. Accordingly, the observed improvements cannot be attributed solely to the specific effects of ISF-NF.

Future controlled studies with longer baseline phases, formal testing of dose–response relationships, and integration of objective neurophysiological measures (e.g., EEG signal analyses or autonomic measures) are needed to clarify mechanisms and strengthen causal inference. In the present study, pre–post EEG data were collected but are reserved for a separate follow-up study, allowing for a more focused and methodologically appropriate analysis. The current study was not designed to assess within-session neurofeedback learning or EEG regulation dynamics. Future research should therefore prioritize designs that enable systematic examination of EEG changes both within and across sessions, in order to determine whether ISF neurofeedback is associated with measurable learning effects and target engagement. Such approaches are critical for establishing neurofeedback as a mechanism-based intervention and for characterizing learning curves, as emphasized in prior work (Enriquez-Geppert et al., 2017). Advanced analytic approaches capable of modeling multimodal sleep data may further enhance understanding of individualized regulatory trajectories.

## 5 Conclusion

This exploratory SCED study conducted in primary care suggests that ISF-NF may be associated with improvements in perceived sleep quality for a subset of individuals with insomnia. Within-person analyses revealed heterogeneous yet interpretable change trajectories, suggesting that individualized parameter adjustment may play a central role in adaptive ISF-NF protocols.

Objective wearable-derived data did not uniformly align with changes in total sleep time but indicated that shifts in sleep organization, including improved consolidation and timing, may represent relevant dimensions of change. These findings reinforce the multidimensional nature of sleep health and the importance of integrating subjective and objective measures when evaluating treatment response.

ISF-NF appeared to be feasible and well tolerated in a primary care context, with high alliance and satisfaction ratings and few reported adverse effects. However, given the exploratory design, limited sample size, and partial attenuation of group-level questionnaire effects at follow-up, the findings should be interpreted cautiously.

Future research should prioritize controlled designs, replication across settings, and systematic examination of dose–response relationships and maintenance of effects. Integration of multimodal neurophysiological and psychosocial measures may further clarify mechanisms and inform the refinement of individualized ISF-NF parameter adjustment strategies.

## Data Availability

The datasets generated and analyzed during the current study are not publicly available due to privacy and ethical restrictions. De-identified data relevant to the reported analyses may be made available by the corresponding author upon reasonable scientific request and subject to applicable ethical and legal requirements.

## Footnotes

1
https://singlecaseresearch.org/calculators/tau-u/
