# Correction: Infra-slow EEG neurofeedback for insomnia: a single-case experimental study in primary care

**DOI:** 10.3389/fnhum.2026.1961428

**Published:** 2026-08-21

**Authors:** Frontiers Production Office

**Affiliations:** Frontiers Media SA, Lausanne, Switzerland

**Keywords:** activity tracker, brain activity, complex interventions, EEG-biofeedback, infraslow oscillations, insomnia, primary care, sleep health

An erroneous affiliation [10. sensLab, Division of Digital Development and Innovation, Sahlgrenska University Hospital, Gothenburg, Sweden] was added to the affiliations list. This affiliation has now been removed.

The original version of this article has been updated.

