# Supplementary material for: Infra-slow EEG neurofeedback for insomnia: a single-case experimental study in primary care
Source: Front Hum Neurosci. 2026 Jul 16;20:1832178. doi: 10.3389/fnhum.2026.1832178 (PMC13422157; doi:10.3389/fnhum.2026.1832178)
Supplement: Supplementary file 1 [file Data_Sheet_1.PDF]

Supplementary Material

Appendix Table of Contents

A Quantitative Analyses (BSRSQA, Tau-U, Z-Scores)

Appendix A1. Median BSRSQA Scores by Phase and Participant

B. Diagnostic Instruments and Self-Report Measures

Appendix B1. PSQI, PHQ-9, and GAD-7 Scores Across Timepoints by Participant

Appendix B2. Negative Effects Questionnaire (NEQ) – Endorsed Items and Attributions

Appendix B3. Client Satisfaction Questionnaire (CSQ-8) – Summary of Ratings

C. Objective Sleep Data (Wearable-Derived)

Appendix C1. Wearable Data Availability and Completeness

Appendix C2.1. Actogram-Style Sleep Timing and Total Sleep Time (TST)

Appendix C2.2. Single-Case Descriptive Overview of Total Sleep Time (TST)

Appendix C3.1 Single-Case Tau Analyses for Total Sleep Time (TST)

Appendix C4.1. Participant-Level Visual Interpretation of Actogram-Based Sleep structure (Figure 4)

Appendix C5.1 Actogram-Style Visualization of Stage-Based Awakenings Across 140 days (18:00–18:00).

Appendix C5.2 Percentage of Awake Time During the Sleep Period (PADS) Across 140 days (18:00–18:00)

Appendix C5.3. Interpretation of PADS Trends Across Participants

Appendix C6. Integrated Overview of Subjective and Objective Sleep Outcomes

D. Participant Inclusion and Case Profiles

Appendix D1. Overview of Data Completeness and Inclusion Status by Participant

Appendix D2. Rationale for Inclusion in Main Text vs. Appendix

Appendix D3. Visual BSRSQA Analysis – Participants Not Included in Main Text

E. Materials and Surveys

Appendix E1. Symptom Monitoring Checklist: 24- and 48-Hour Post-Training Feedback

Appendix E2. Subjective Sleep Rating (BSRSQA)

F. Methodological Transparency

Appendix F1. Alignment With the CRED-nf Checklist (Ros et al., 2020)

Appendix F2. Ethical Approval

Appendix

Appendix A1. Median BSRSQA Scores by Phase and Participant

Participant	Baseline	Non-optimal	Optimal
P01	8.5	11.0	16.0
P02	18.0	25.0	27.0
P03	20.0	20.0	
P04	15.0	18.5	
P05	20.0		21.0
P06	11.0	15.0	20.0
P07	15.0	16.0	19.0
P08	11.0	13.0	
P09	18.0	22.0	22.0
P10	15.0	14.0	12.0

Note. Median values are based on available daily BSRSQA ratings within each phase. Empty cells indicate that a given phase could not be defined or that insufficient data were available. For P03, an individualized optimal ISF-NF parameter setting was not established during the intervention. For P05, the individualized optimal parameter setting was identified early, resulting in minimal separation between non-optimal and optimal phases. For P04, the non-optimal phase median (18.5) was based on only a few daily ratings before daily reporting was discontinued and should therefore be interpreted with caution. For P08, the intervention was discontinued before an individualized optimal parameter setting could be established.

B. Diagnostic Instruments and Self-Report Measures

Appendix B1. PSQI, PHQ-9, and GAD-7 Scores Across Timepoints by Participant

Individual scores on the Pittsburgh Sleep Quality Index (PSQI), Patient Health Questionnaire-9 (PHQ-9), and Generalized Anxiety Disorder-7 (GAD-7) at baseline (Pre), post-intervention (Post), and 3-month follow-up (3M). Raw scores are presented together with clinical classifications based on established cut-off values. Higher scores indicate greater symptom severity. Dashes indicate missing data due to incomplete assessment at the corresponding time point. The table is provided to illustrate individual trajectories and clinical heterogeneity and was not subjected to inferential group-level statistical testing.

Participant	PSQI_Pre	PSQI_Post	PSQI_3M	PHQ9_Pre	PHQ9_Post	PHQ9_3M	GAD7_Pre	GAD7_Post	GAD7_3M
P01	19 (Clinical)	15 (Clinical)	17 (Clinical)	13 (Moderate)	3 (Minimal)	4 (Minimal)	5 (Mild)	2 (Minimal)	5 (Mild)
P02	11 (Clinical)	4 (Non-clinical)	8 (Clinical)	5 (Mild)	2 (Minimal)	2 (Minimal)	1 (Minimal)	0 (Minimal)	0 (Minimal)
P03	8 (Clinical)	–	6 (Clinical)	2 (Minimal)	–	7 (Mild)	0 (Minimal)	–	0 (Minimal)
P04	12 (Clinical)	6 (Clinical)	4 (Non-clinical)	11 (Moderate)	5 (Mild)	19 (Moderately severe)	2 (Minimal)	6 (Mild)	3 (Minimal)
P05	11 (Clinical)	8 (Clinical)	10 (Clinical)	5 (Mild)	1 (Minimal)	2 (Minimal)	2 (Minimal)	0 (Minimal)	0 (Minimal)
P06	19 (Clinical)	11 (Clinical)	17 (Clinical)	19 (Moderately severe)	10 (Moderate)	21 (Severe)	4 (Minimal)	2 (Minimal)	13 (Moderate)
P07	–	5 (Non-clinical)	10 (Clinical)	15 (Moderately severe)	10 (Moderate)	6 (Mild)	12 (Moderate)	7 (Mild)	13 (Moderate)
P08	16 (Clinical)	–	–	14 (Moderate)	–	–	11 (Moderate)	–	–
P09	14 (Clinical)	9 (Clinical)	14 (Clinical)	9 (Mild)	6 (Mild)	14 (Moderate)	14 (Moderate)	3 (Minimal)	3 (Minimal)

P10	15 (Clinical)	14 (Clinical)	13 (Clinical)	10 (Moderate)	8 (Mild)	10 (Moderate)	2 (Minimal)	0 (Minimal)	3 (Minimal)

Appendix B2. Negative Effects Questionnaire (NEQ) – Endorsed Items and Attributions

Negative effects endorsed on the Negative Effects Questionnaire (NEQ)

Reported symptom (item #)	Reported effect, n	Degree of effect	Treatment related effect, n
Increased sleep problems (1)	1	Moderately	1
Increased stress (2)	1	Slightly	0
Increased anxiety (3)	0		0
Increased worry (4)	0		0
Felt more dejected (5)			
Increased suicidal ideation (15)	1	Slightly	1
Increased sadness (9)	1	Slightly	0
Increased unpleasant feelings (11)	1	Slightly	1
Increased unpleasant memories (13)	0		0
**Hopelessness**			
Increased hopelessness (6)	0		0
I felt that the issue I was looking for help with got worse (12)	0		0
I stopped thinking that things could get better (17)	0		0
Increased perception problem cannot get better (18)	1	Moderately	1

I stopped thinking help was possible (19)	0		0
**Stigma**			
Increased fear people would find out about treatment (14)	1	Slightly	1
Increased shame of having treatment (16)	0		0
**Personal failure**			
Decreased self-esteem (7)	0		0
Lost faith in my self (8)	0		0
I felt less competent (10)	0		0
**Therapy-level effects**			
**Dependency**			
Developed dependency on treatment (20)	1	Not at all	1
Developed dependency on therapist (21)	0		0
**Quality**			
Did not understand treatment (22)	3	Not at all, Slightly, Slightly	3
Did not understand therapist (23)	2	Not at all, Slightly	2
Did not have confidence in treatment (24)	1	Not at all	1
Did not have confidence in therapist (25)	1	Not at all	1
Treatment did not produce results (26)	0		0
Treatment expectations were not fulfilled (27)	0		0
Therapist expectations were not fulfilled (28)	0		0

Treatment quality was poor (29)	0		0
Treatment did not suit (30)	0		0
No close relationship with therapist (31)	0		0
Treatment was not motivating (32)	0		0

Appendix B3. Client Satisfaction Questionnaire (CSQ-8) – Summary of Ratings

Client Satisfaction Questionnaire (CSQ-8): mean response scores and percentages indicating each response (n = 8)

Questionnaire item	Mean	4	3	2	1
How would you rate the quality of the service you received?	3.25	25% Excellent	75% Good	0% Fair	0% Poor
To what extent has our service met your needs?	3	12.5% Yes definitely	75% Yes generally	12.5% No not really	0% No definitely not
Did you get the kind of service you wanted?	2.62	12.5% Almost all met	37.5% Most met	50% Only a few met	0% None met
If a friend were in need of similar help would you recommend our service?	3.38	37.5% Yes definitely	62.5% Yes I think so	0% No I do not think so	0% Definitely not
How satisfied are you with the amount of help you received?	3.38	37.5% Very satisfied	62.5% Mostly satisfied	0% Indifferent	0% Quite dissatisfied
Have the services you received helped you to deal more effectively with your problems?	3.25	37.5% Yes a great deal	50% Yes somewhat	12.5% No did not help	0% No made it worse
In an overall sense, how satisfied are you with the service you have received?	3.38	37.5% Very satisfied	62.5% Mostly satisfied	0% Indifferent	0% Quite dissatisfied
If you were seeking help again, would you come back to our service?	3.25	37.5% Yes definitely	50% Yes, I think so	12.5% No I do not think so	0% No definitely not

Appendix C. Objective Sleep Data (Wearable-Derived)

Appendix C presents complementary analyses of wearable-derived sleep outcomes, including data availability (C1), actogram-based sleep timing and total sleep time (C2–C3), sleep structure and fragmentation (C4–C5), and an integrated participant-level overview of subjective and objective sleep outcomes (C6).

Appendix C1. Wearable Data Availability and Completeness

Participant	Total study days	Nights with valid data	Nights missing	Missing (%)
**P01**	97	83	14	14.4
**P02**	113	112	1	0.9
**P04**	101	74	27	26.7
**P05**	96	95	1	1.0
**P06**	113	113	0	0.0
**P07**	133	98	35	26.3
**P08**	87	80	7	8.0
**P09**	113	113	0	0.0
**P10**	139	138	1	0.7

Note on data completeness.
Data completeness varied across participants due to temporary non-wear, synchronization issues, and external circumstances affecting adherence. For two participants (P04 and P07), higher proportions of missing wearable data were observed. These interruptions were unrelated to the intervention procedures themselves.

Appendix C2.1. Actogram-Style Sleep Timing and Total Sleep Time (TST)

Black segments represent recorded sleep episodes. Blue dashed vertical lines mark intervention onset, and green dotted lines indicate the day on which an individualized optimal ISF-NF parameter setting was established, where applicable.

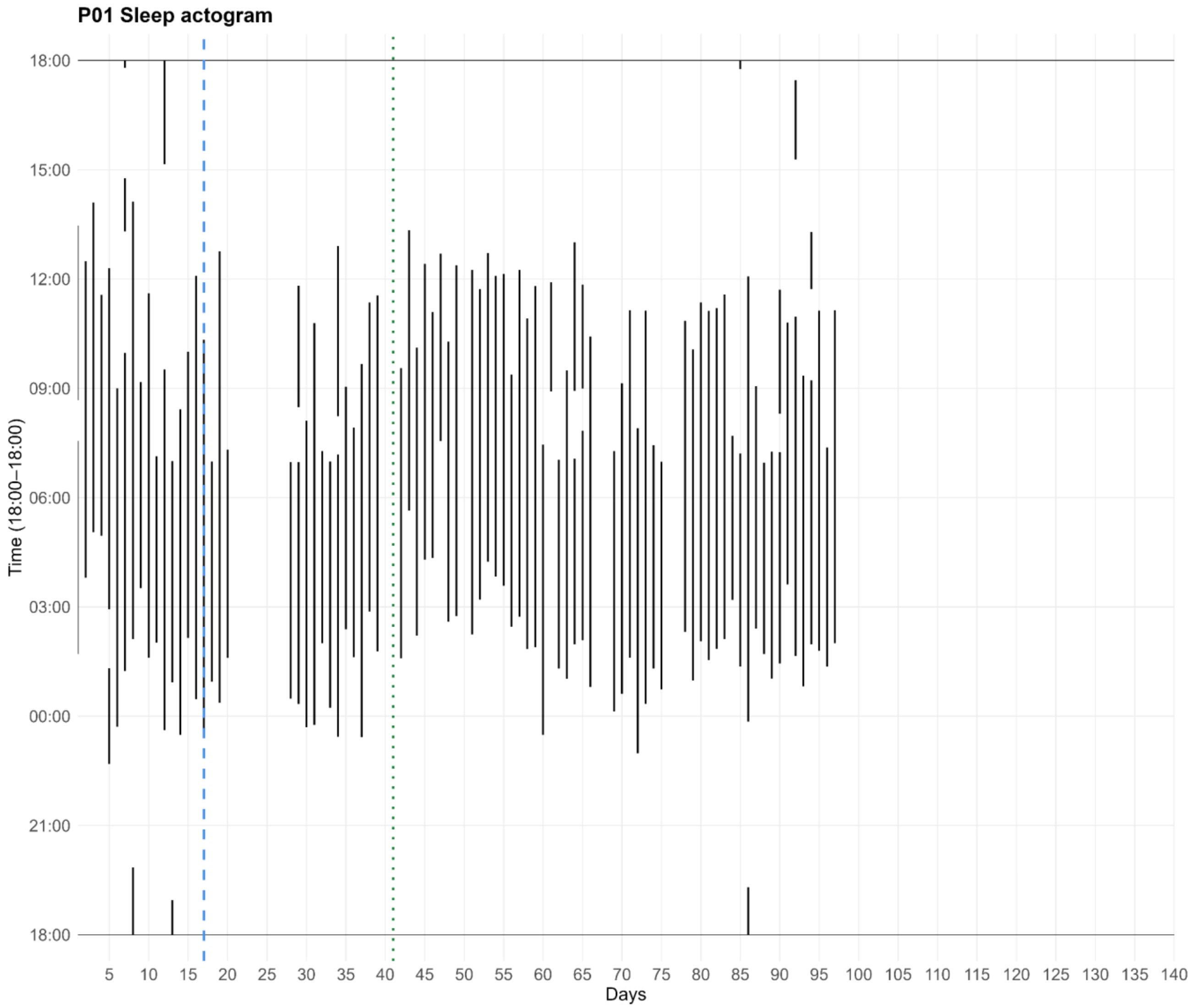

Figure C2.1a. Participant P01

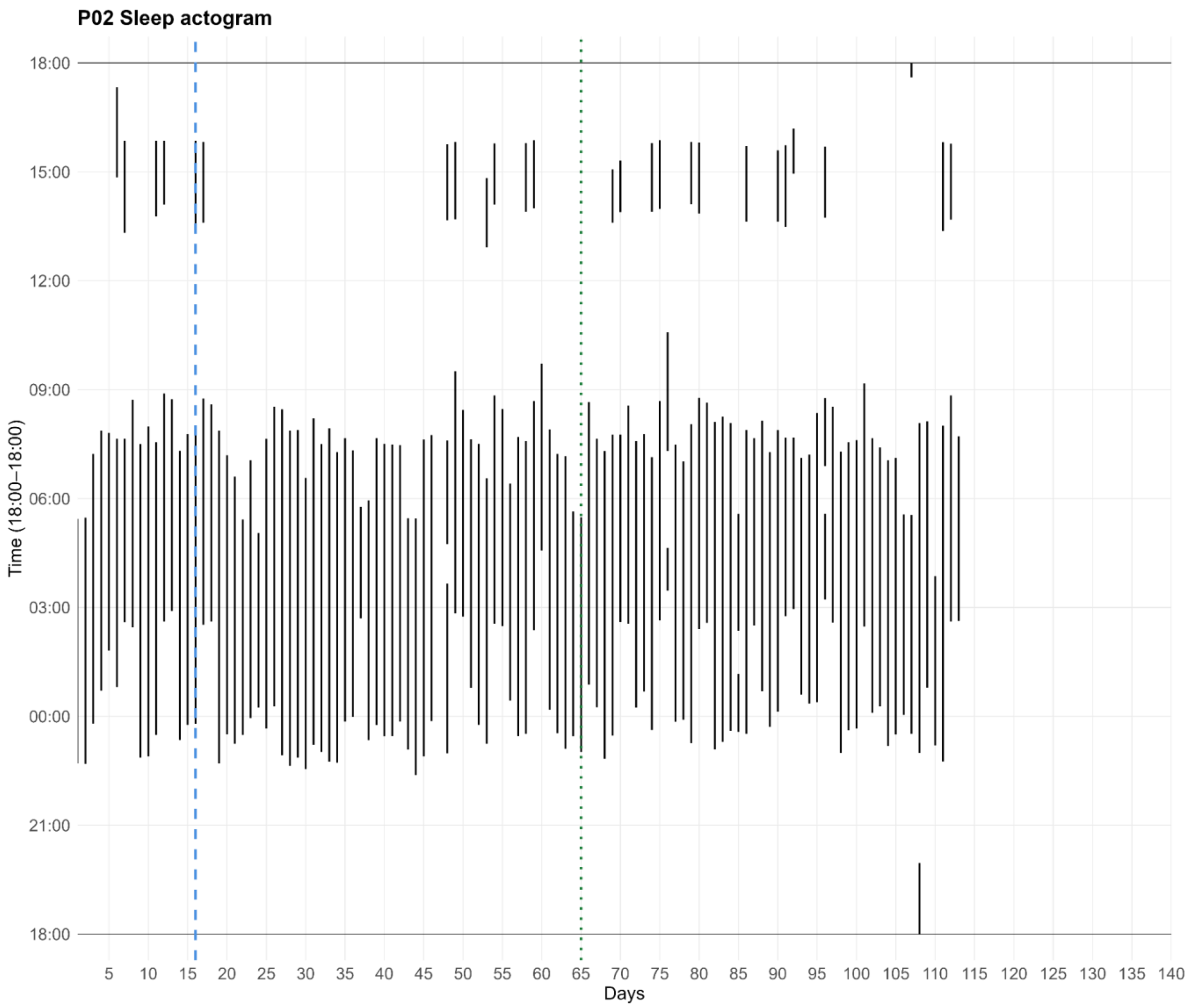

Figure C2.1b. Participant P02

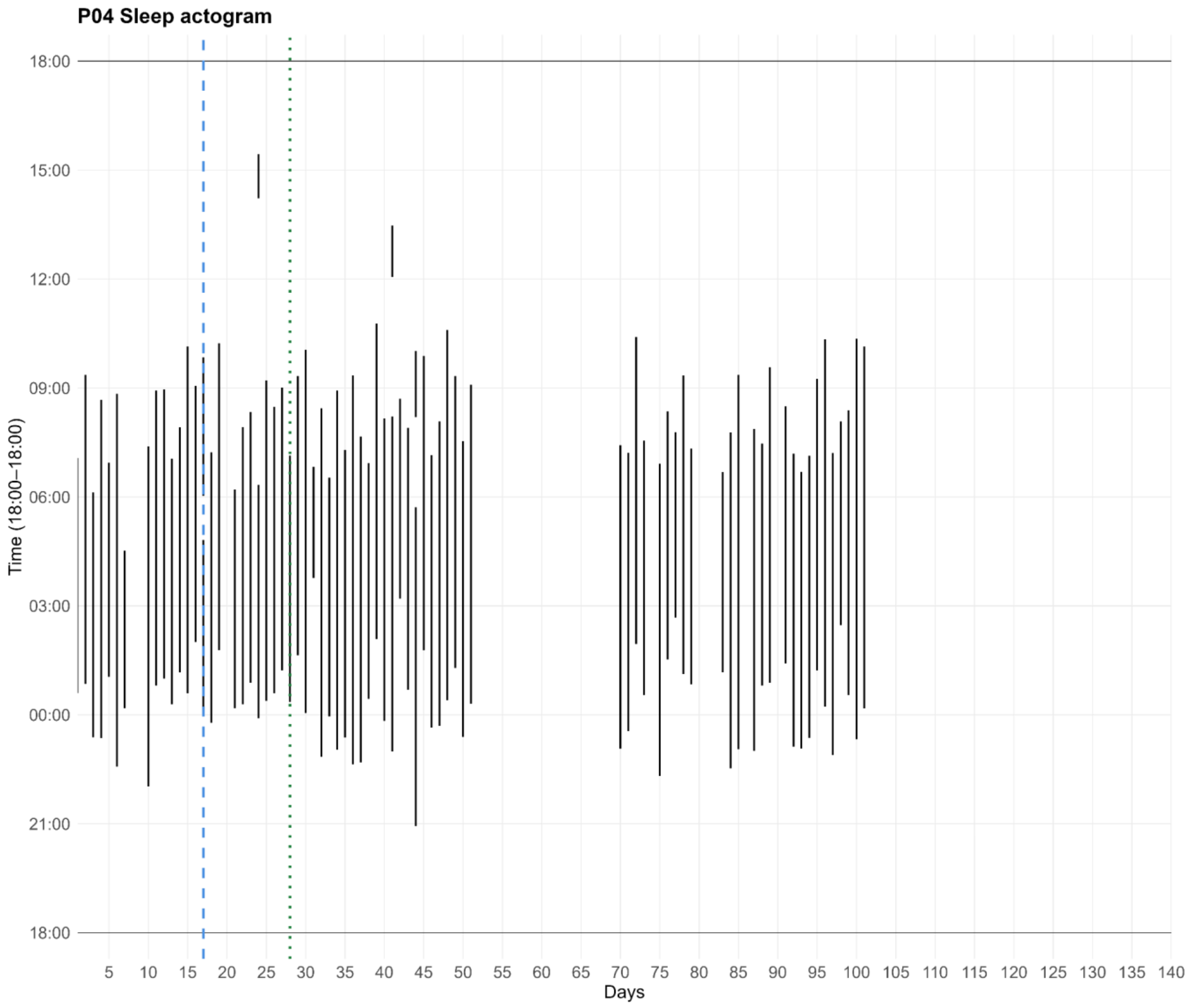

Figure C2.1c. Participant P04

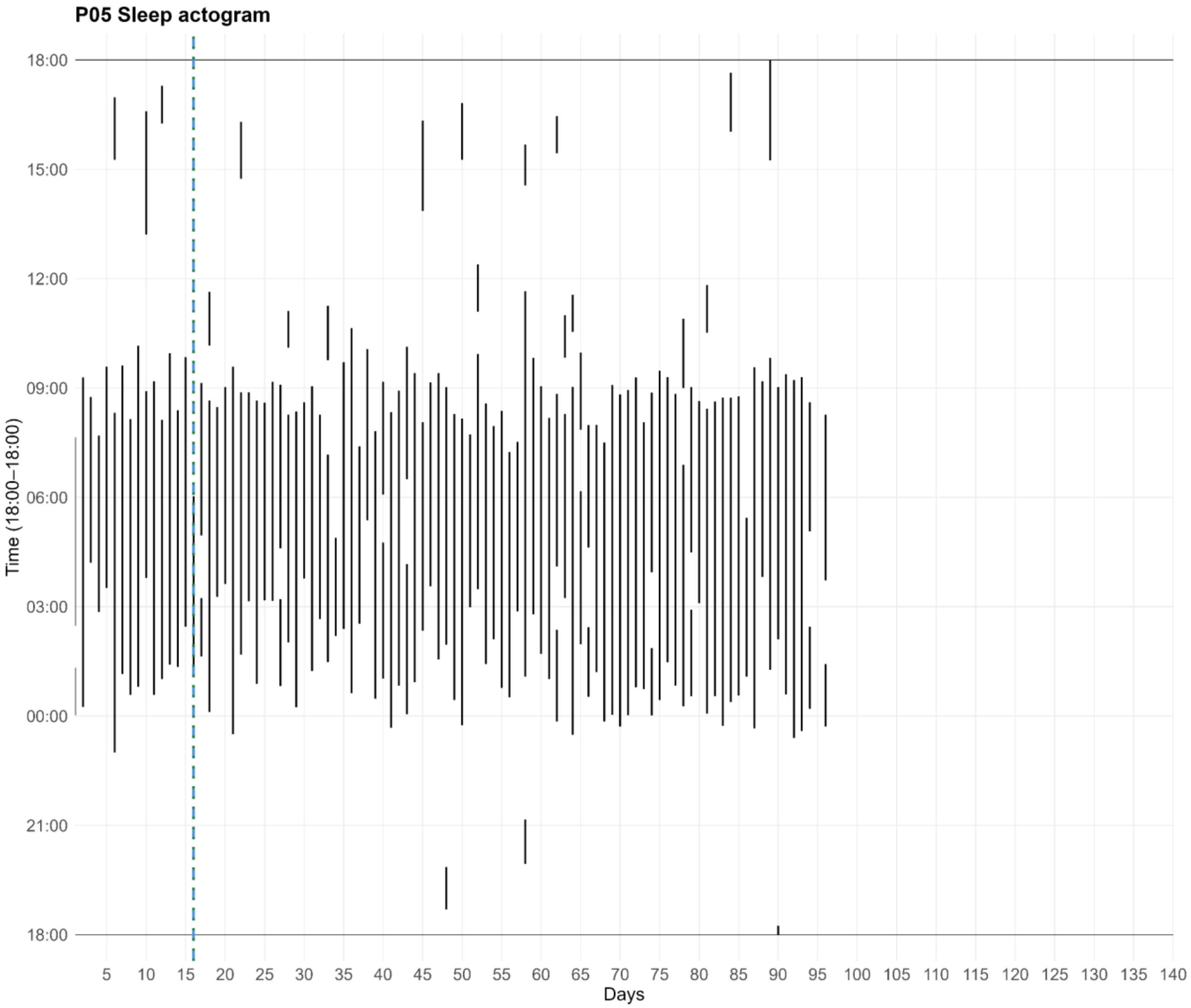

Figure C2.1d. Participant P05

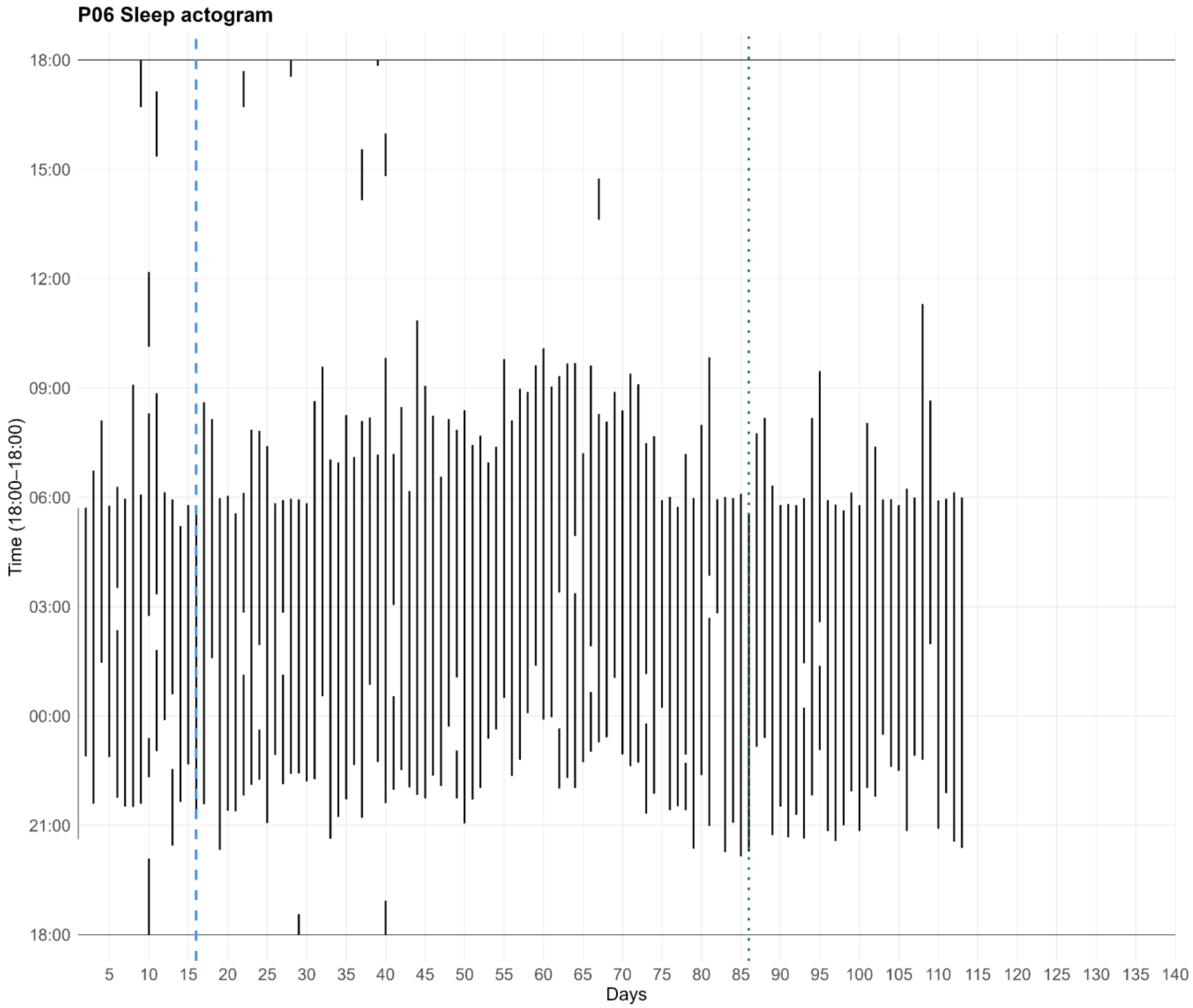

Figure C2.1e. Participant P06

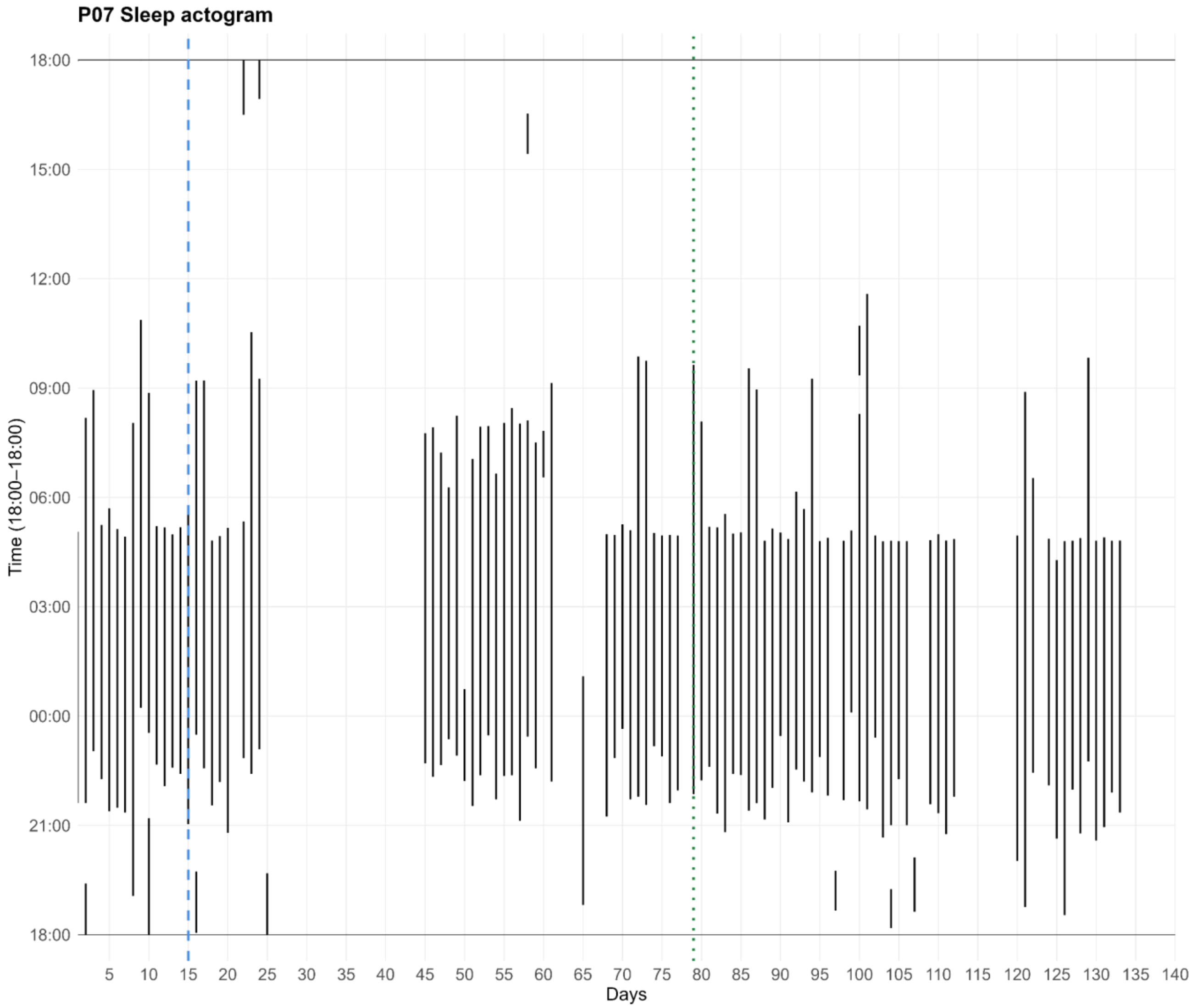

Figure C2.1f. Participant P07

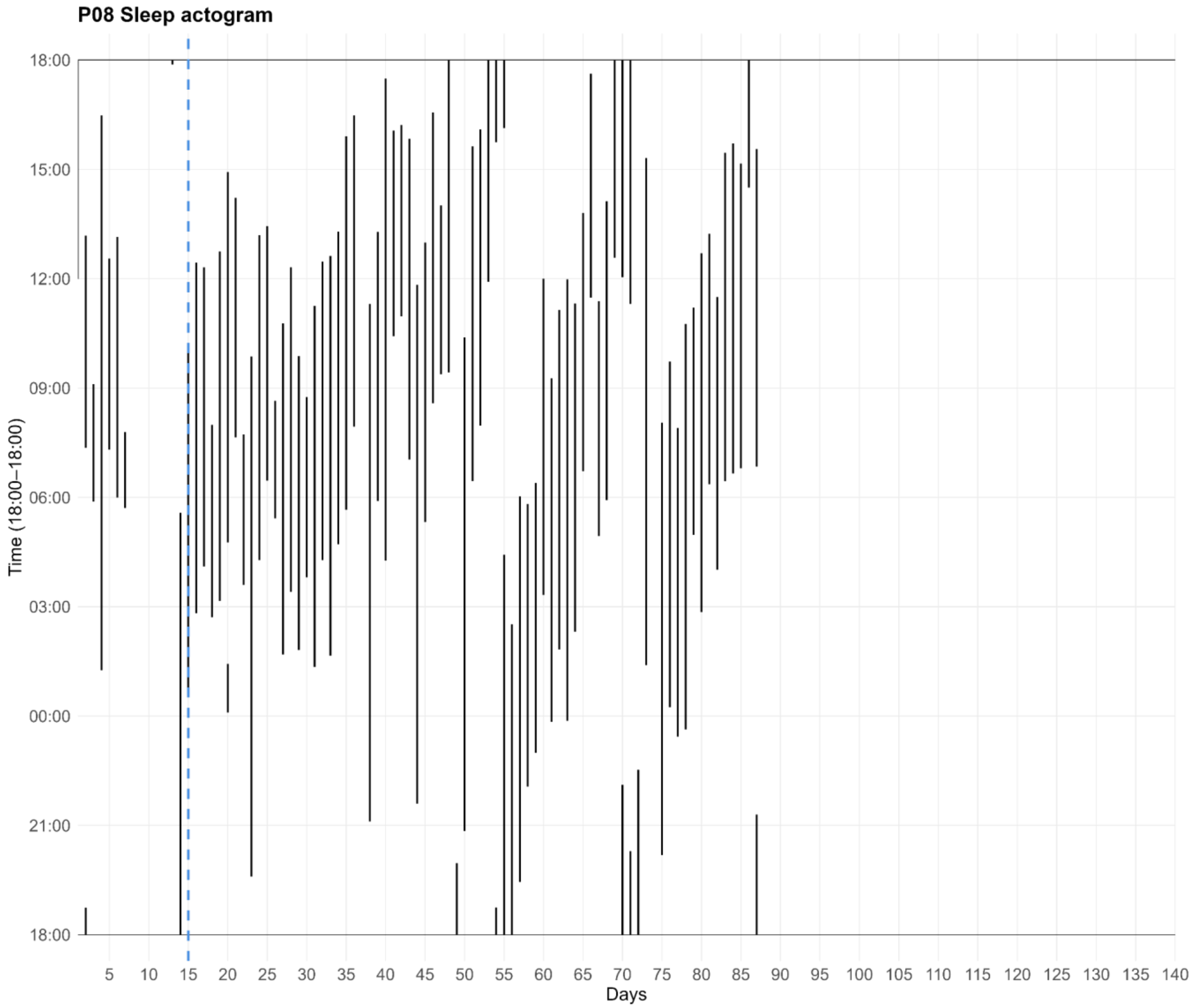

Figure C2.1g. Participant P08

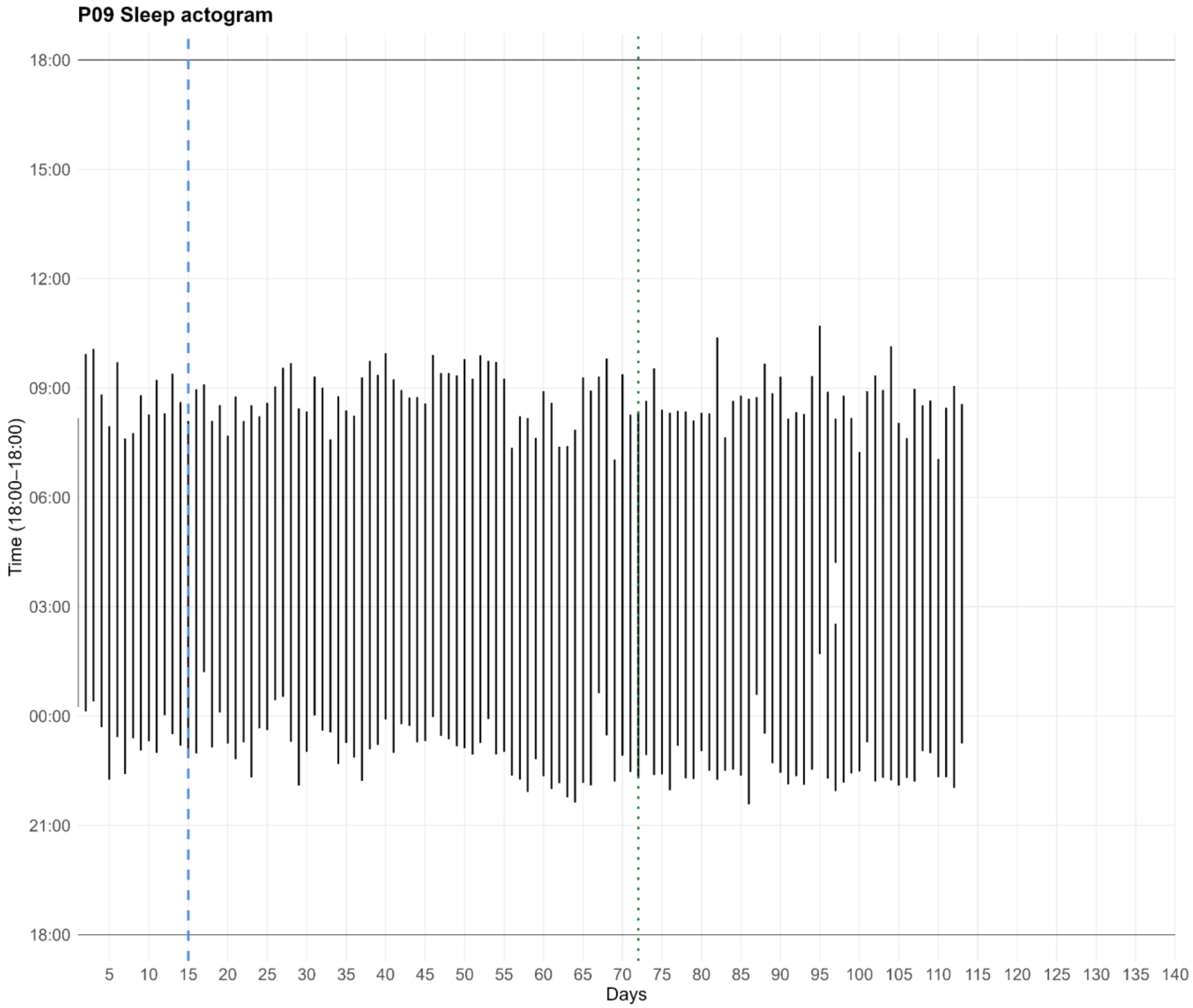

Figure C2.1h. Participant P09

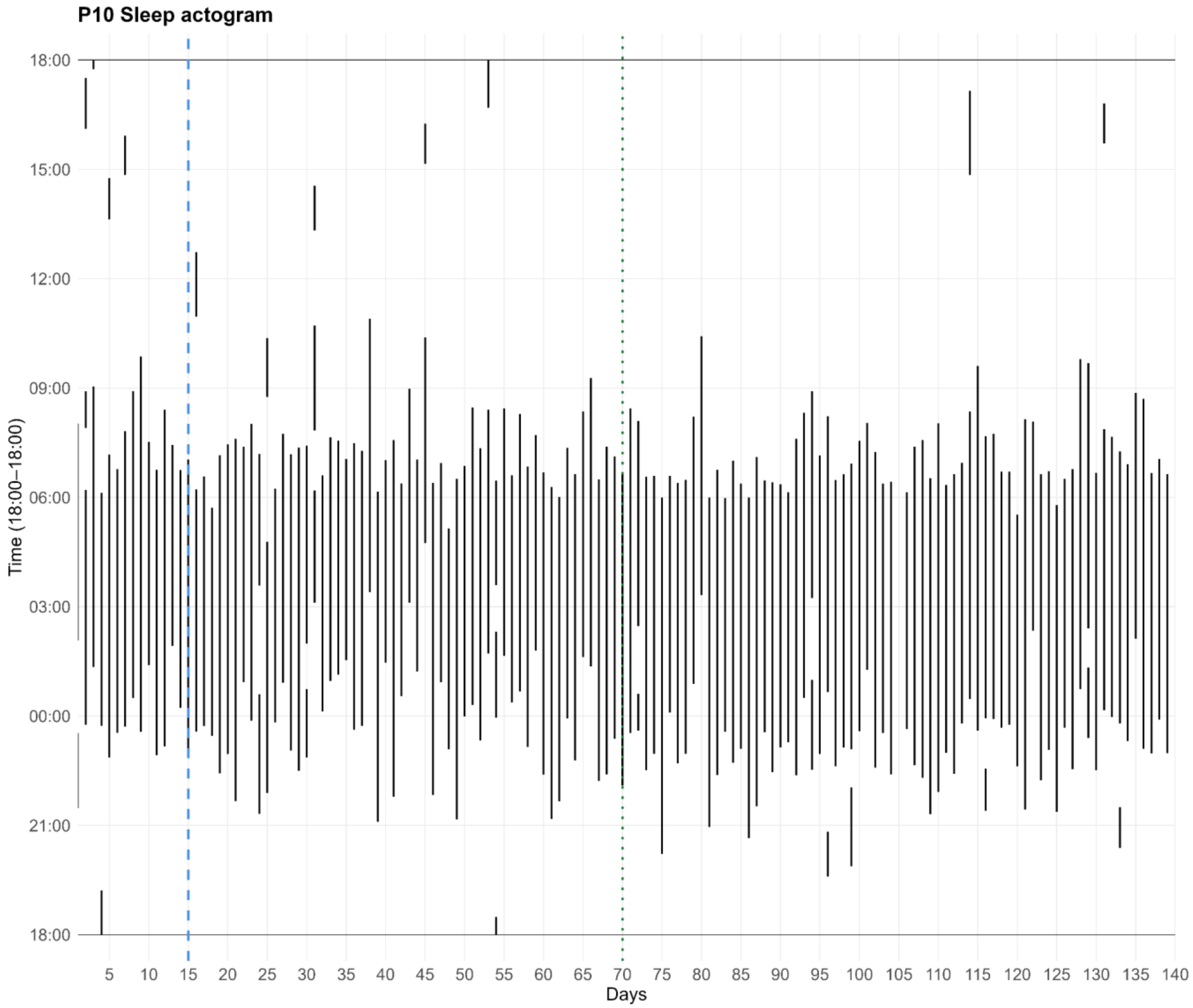

Figure C2.1i. Participant P10

Note. Visual inspection of sleep timing plots revealed marked inter-individual variability. Some participants showed clear improvements in sleep consolidation or circadian organization over the intervention period, whereas interpretation for others was complicated by contextual factors such as shift work, travel, or irregular daily routines. These visualizations are therefore presented to support interpretation of quantitative analyses rather than as independent outcome measures.

Appendix C2.2. Single-Case Descriptive Overview of Total Sleep Time (TST)

Participant	TST pattern during baseline	TST pattern during intervention	Tau (B vs I)	Direction	Descriptive interpretation

**P01**	Variable	Slightly lower and less variable	−0.26 (ns)	↓	Reduced total sleep time despite subjective improvement
**P02**	Variable	Slightly lower	−0.13 (ns)	↓	Minor reduction in total sleep time; interpretation complicated by night work and on-call shifts
**P03**	—	—	—	n/a	Objective TST data unavailable
**P04**	Variable	Comparable to baseline	−0.05 (ns)	→	Stable total sleep time
**P05**	Variable	Comparable to baseline overall, slightly higher toward the end	−0.06 (ns)	→	Stable total sleep time with a late-phase increase
**P06**	Variable	Slightly higher	0.17 (ns)	↑	Modest increase in total sleep time
**P07**	Short	Longer nights during intervention	0.45 (ns)	↑	Moderate increase in total sleep time
**P08**	Highly variable	Slightly higher (limited exposure)	0.28 (ns)	↑	Increased total sleep time with limited intervention exposure
**P09**	Relatively stable	Gradual increase	0.33 (ns)	↑	Moderate increase in total sleep time
**P10**	Short and variable	Slightly lower overall, with a late-phase increase	−0.15 (ns)	↓	Reduced total sleep time overall, despite a late-phase increase

Note. Total sleep time (TST) was derived from wearable sleep episode data and summarized at the daily level. Tau values reflect single-case comparisons between baseline and intervention phases (see Appendix C3). All Tau estimates in this table were non-significant ($p > .05$) and are therefore interpreted descriptively. Directional labels indicate the predominant trend across the intervention

phase and should be interpreted in conjunction with actogram-based sleep structure (Appendix C4) and nocturnal wakefulness indices (PADS; Appendix C5).

Appendix C3.1 Single-Case Tau Analyses for Total Sleep Time (TST)

Participant	Comparison	Tau	Z	p-value	90% CI	Direction
P01	**B vs I**	**−0.26**	**−1.11**	**.268**	**−0.64 to 0.13**	**↓ TST**
P02	**B vs I**	**−0.13**	**−0.53**	**.595**	**−0.52 to 0.27**	**↓ TST**
P04	**B vs I**	**−0.05**	**−0.16**	**.873**	**−0.51 to 0.42**	**Stable**
P05	**B vs I**	**−0.06**	**−0.26**	**.797**	**−0.45 to 0.33**	**Stable**
P06	**B vs I**	**0.17**	**0.74**	**.458**	**−0.21 to 0.56**	**↑ TST**
P07	**B vs I**	**0.45**	**1.48**	**.139**	**−0.05 to 0.96**	**↑ TST**
P08	**B vs I**	**0.28**	**0.92**	**.358**	**−0.22 to 0.79**	**↑ TST**
P09	**B vs I**	**0.33**	**1.31**	**.190**	**−0.08 to 0.74**	**↑ TST**
P10	**B vs I**	**−0.15**	**−0.62**	**.537**	**−0.57 to 0.26**	**↓ TST**

Note. Positive Tau values indicate increased total sleep time during the intervention phase, whereas negative values indicate reduced total sleep time. Confidence intervals crossing zero reflect substantial variability and underscore the heterogeneity of individual responses.

Appendix C4.1. Participant-Level Visual Interpretation of Actogram-Based Sleep Structure (Figure 4).

Participant	Baseline fragmentation	Baseline timing regularity	Change during intervention	Post-optimization fragmentation	Post-optimization timing	Descriptive summary

P01	High fragmentation	Irregular	Gradual reduction in fragmentation and improved timing	Reduced	Improved	Reduced fragmentation and improved timing regularity
P02	Moderate fragmentation	Irregular	Minimal change	No clear change	No clear change	Limited structural change
P03	—	—	—	—	—	Objective actogram data unavailable
P04	Low fragmentation	Irregular	Gradual improvement in timing	Stable	Improved	Improved timing regularity
P05	High fragmentation	Irregular	Gradual reduction in fragmentation	Reduced	Improved	Reduced fragmentation and improved timing
P06	High fragmentation	Irregular	Reduction in fragmentation	Reduced	Improved	Reduced fragmentation and improved timing
P07	Moderate fragmentation	Irregular	Reduction in fragmentation	Reduced	Improved	Reduced fragmentation and improved timing
P08	High fragmentation and irregular timing	Highly irregular	Initial improvement followed by relapse	n/a	n/a	Initial structural improvement followed by dropout

P09	Low fragmentation	Relatively regular	Improved timing without consolidation change	Stable	Improved	Improved timing only
P10	High fragmentation	Irregular	Reduced fragmentation	Reduced	Improved	Reduced fragmentation and improved timing

Appendix C5.1 Actogram-Style Visualization of Stage-Based Awakenings Across 140 Days (18:00–18:00).

Black segments indicate wake periods derived from Fitbit sleep-stage classification. Dark grey dashed vertical lines mark intervention onset; grey dotted lines mark the day on which an individualized optimal ISF-NF parameter setting was established, where applicable. Participant P04 is excluded due to insufficient sleep-stage data quality.

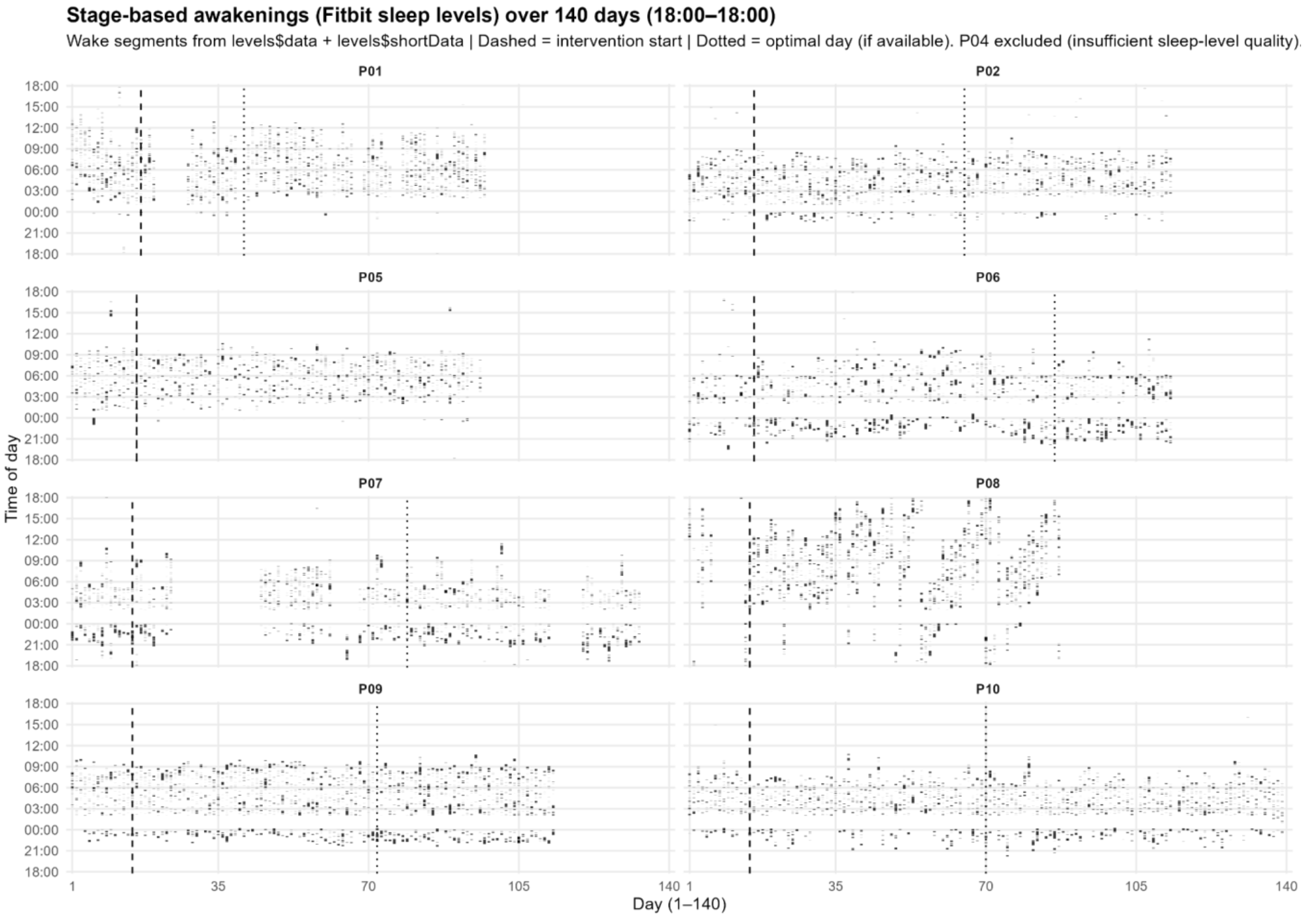

Stage-based awakenings represent brief wake periods detected by the device's sleep-staging algorithm and may capture micro-awakenings not apparent from episode-based sleep segmentation alone. Visual patterns should be interpreted cautiously, as detection is device- and algorithm-dependent and may vary with data quality.

Appendix C5.2 Percentage of Awake Time During the Sleep Period (PADS) Across 140 Days (18:00–18:00).

PADS was computed per day as: **PADS (%) = 100 × awake_stage_minutes / (awake_stage_minutes + TST_minutes)**

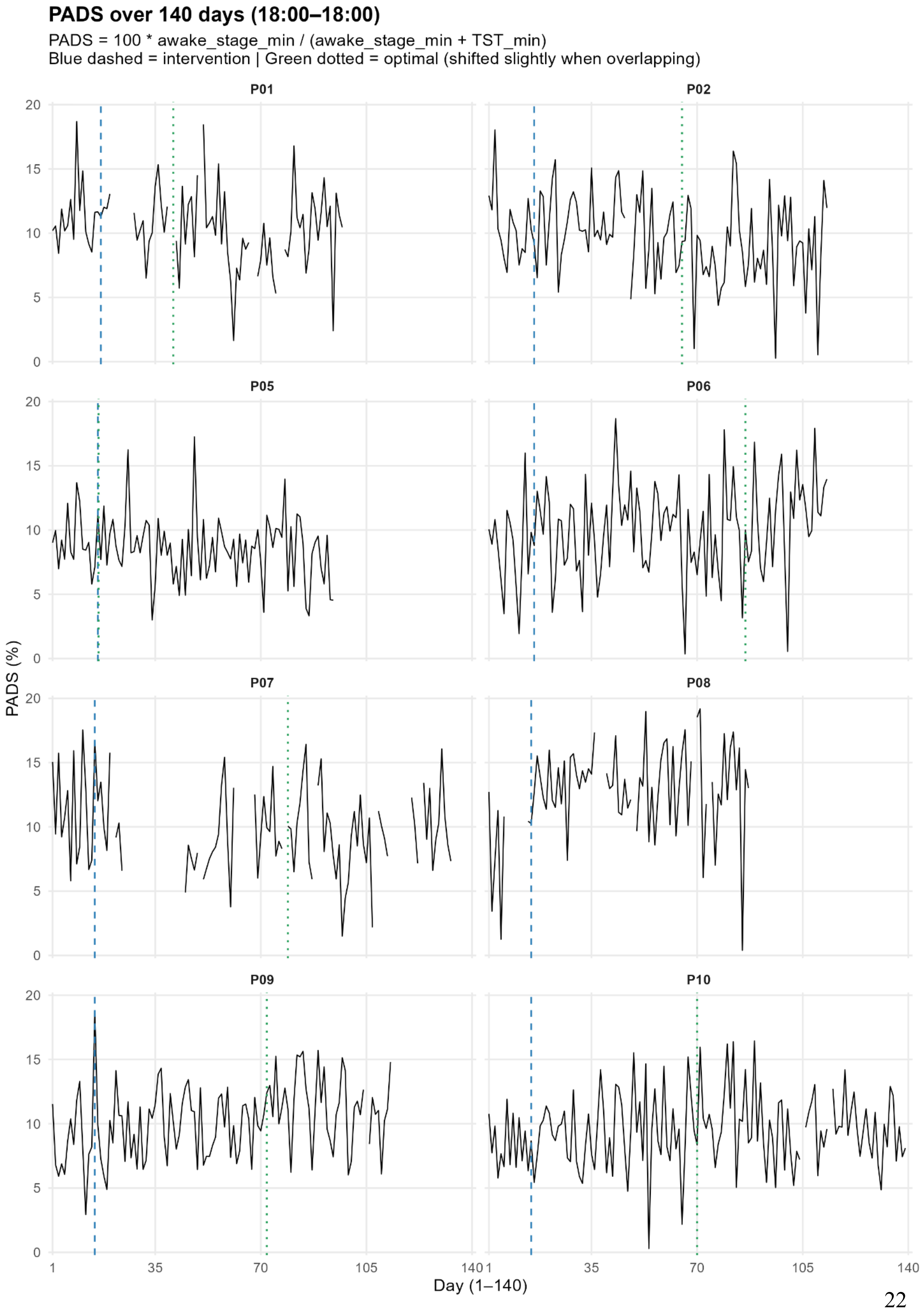

PADS over 140 days (18:00–18:00)
PADS = 100 * awake_stage_min / (awake_stage_min + TST_min)
Blue dashed = intervention | Green dotted = optimal (shifted slightly when overlapping)
P01
P02
P05
P06
P07
P08
P09
P10
PADS (%)
20
15
10
5
0
1
35
70
105
140
Day (1–140)

Note. Vertical markers indicate intervention onset (blue dashed) and the day on which an individualized optimal **parameter setting** was established (green dotted), where applicable. Participant P04 is excluded due to insufficient sleep-stage data quality. PADS provides a standardized, proportional index of within-sleep wakefulness and complements episode-based fragmentation measures. Given reliance on proprietary sleep-stage classification, results are interpreted descriptively.

Appendix C5.3. Interpretation of PADS Trends Across Participants

Participant	PADS trend	Interpretation
**P01**	↓	Small and variable reduction in nocturnal wakefulness
**P02**	↓	Trend toward reduced nocturnal wakefulness
**P03**	n/a	No wearable sleep-stage data available
**P04**	n/a	Insufficient sleep-stage data
**P05**	↓	Small and variable reduction in nocturnal wakefulness
**P06**	↑	Small increase in nocturnal wakefulness
**P07**	↓	Small and variable reduction in nocturnal wakefulness
**P08**	↑	Increased nocturnal wakefulness during limited intervention exposure
**P09**	→	Mixed pattern with no clear change in nocturnal wakefulness
**P10**	↑	Increased variability and nocturnal wakefulness compared to baseline

Note. Participant P04 was excluded from stage-based awakening analyses due to insufficient sleep-stage data quality. Sleep staging information (levels data) was largely missing, rendering awake-stage estimates unreliable. The corresponding plots are therefore presented for descriptive and exploratory purposes only, to support transparency and facilitate interpretation of how fragmentation-related indices were derived from the available wearable outputs.

Appendix C6. Integrated Overview of Subjective and Objective Sleep Outcomes

Participant	Subjective sleep (visual BSRSQA)	Subjective sleep (Tau-U)	TST change (wearable)	Sleep consolidation	Sleep timing	PADS (wakefulness)	Timing relative to optimization	Overall convergence	Notes
**P01**	Clear improvement post-optimization	↑↑ (significant)	↓ (ns)	Improved	Improved	↓	Post-optimization	Moderate	
**P02**	Early improvement, sustained	↑↑ (significant)	↓ (ns)	No clear change	No clear change	↓	Early, not clearly linked	Low–Moderate	
**P03**	No visual change	→ (ns)	n/a	n/a	n/a	n/a	n/a	n/a	Wearable data collected but lost due to accidental deletion (n/a across metrics)
**P04**	Insufficient BSRSQA data for SCED analysis	n/a	→ (ns)	No clear change	Improved	n/a	Gradual, interrupted	Low–Moderate	Perceived sleep improvements reported verbally during sessions (not included in SCED analysis)
**P05**	Gradual improvement	↑ (trend)	→ (ns; late increase)	Improved	Improved	↓	Gradual across intervention	Moderate	
**P06**	Clear and sustained improvement	↑↑ (significant)	↑ (ns)	Markedly improved	Improved	↑	Post-optimization	Moderate–High	
**P07**	Gradual late improvement	↑↑ (significant)	↑ (ns)	Markedly improved	Improved	↓	Post-optimization	High	Sleep apnea identified post-intervention

**P08**	High variability, early dropout	↓ (ns)	↑ (ns; limited exposure)	Initial improvement → relapse	Initial improvement → relapse	↑	Not assessable	Low	Early dropout; limited exposure; signal affected by sweat-related artifacts
**P09**	Gradual stabilization	↑↑ (significant)	↑ (ns)	No clear change	Improved	→	Gradual across intervention	Moderate	
**P10**	Increased stability without quality gain	→ (ns)	↓ (ns; late increase)	Improved	Improved	↑	Post-optimization	Low–Moderate	

Note. Arrows indicate direction of change relative to baseline (↑ improvement, ↓ deterioration, → no clear change). Double arrows (↑↑) indicate statistically significant Tau-U effects ($p < .05$). ns = non-significant.

Appendix D. Participant Inclusion and Case Profiles

Appendix D1. Overview of Data Completeness and Inclusion Status by Participant

Participant ID	Completed Intervention	BSRSQA Data Available / Trend Observed	PSQI Pree - Post Data	PHQ-9 Pree - Post Data	GAD-7 Pree - Post Data	Fitbit Data (Completeness)	Interview Included in Qualitative Analysis	Comment
**P01**	Yes	Yes	Yes	Yes	Yes	Yes	Yes	Fully complete datasets. Included in all analyses.
**P02**	Yes	Yes	Yes	Yes	Yes	Yes	Yes	Fully complete datasets. Included in all analyses.
**P05**	Yes	Yes	Yes	Yes	Yes	Yes	Yes	Fully complete datasets. Included in all analyses.
**P06**	Yes	Yes	Yes	Yes	Yes	Yes	Yes	Fully complete datasets. Included in all analyses.
**P09**	Yes	Yes	Yes	Yes	Yes	Yes	Yes	Fully complete datasets. Included in all analyses.
**P10**	Yes	Yes	Yes	Yes	Yes	Yes	Yes	Fully complete datasets. Included in all analyses.

**P03**	Yes	Yes	Post missing	Post missing	Post missing	No	Yes	Missing posttest data for PSQI, PHQ-9, and GAD-7. Included in visual SCED analysis, excluded from group-level statistics for self-reports. Wearable data collected but lost due to accidental deletion (n/a across metrics)
**P04**	Yes	No	Yes	Yes	Yes	Partial	Yes	Limited BSRSQA data (insufficient for SCED analysis). Fitbit data available but partial. Included in group-level stats but excluded from SCED visual analysis.
**P07**	Yes	Yes	Pree missing	Yes	Yes	Yes	Yes	Missing pretest PSQI. Included in SCED and other analyses where data was complete.
**P08**	No	Limited	Post missing	Post missing	Post missing	Limited	No	P08 discontinued the intervention halfway; insufficient data for SCED and statistical analyses.

Appendix D2. Rationale for Inclusion in Main Text vs. Appendix

Participant ID	**Included in Main Text**	**Reason for Inclusion**	**Comment**
P01	**Yes**	**Complete data, strong responder pattern in BSRSQA and clinical measures**	**Representative case**
P06	**Yes**	**Gradual but stable improvement**	**Useful for showing moderate responder**
P10	**Yes**	**Weak sleep response but meaningful clinical gains (e.g. pain reduction)**	**Shows broader benefit.**
P02	**No**	**Consistent improvements, full data, strong clinical change**	**Illustrative of typical trajectory. Included in appendix for completeness.**
P03	**No**	**Missing posttest clinical data; visual BSRSQA trend available**	**Valuable for visual-only analysis. Included in appendix for completeness.**
P04	**No**	**Almost no BSRSQA data; only partial Fitbit data**	**Limited inclusion possible. Included in appendix for completeness**
P05	**No**	**Complete data but not selected due to redundancy**	**Included in appendix for completeness**
P07	**No**	**Missing pretest PSQI; included in appendix for transparency**	**Diagnosed with severe sleep apnea post-intervention. Included in appendix for completeness.**
P08	**No**	**Dropped out midway; insufficient data for analysis**	**Included in appendix for completeness**
P09	**No**	**Clear gains in both visual and clinical data**	**Strong example of treatment effect. Included in appendix for completeness**

Note. Cases included in the main text were purposefully selected based on data completeness, intervention adherence, and their ability to illustrate distinct response patterns observed in the study (clear improvement, gradual improvement, and limited sleep-specific response). This approach was used to provide illustrative examples of heterogeneous trajectories while maintaining readability of the main manuscript. All remaining participants are presented in Appendix D3 to ensure transparency and allow comparison across the full sample.

Appendix D3. Visual BSRSQA Analysis – Participants Not Included in Main Text

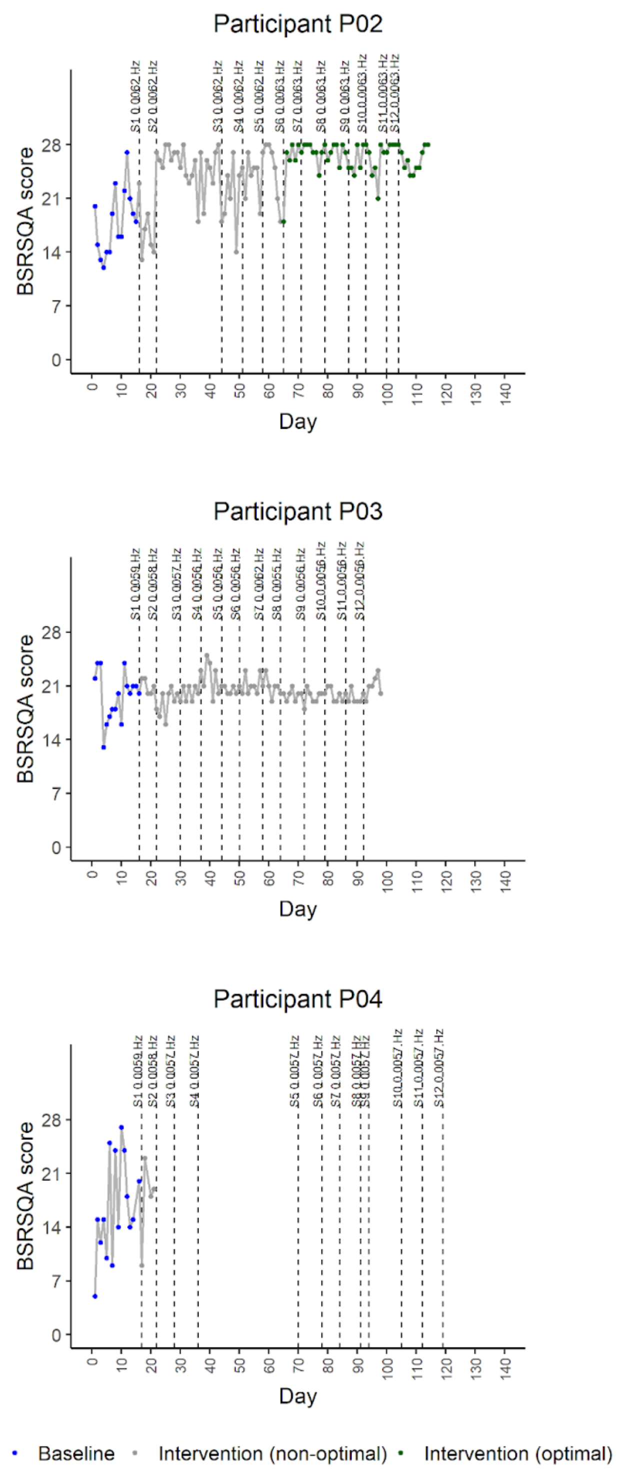

Participant P02. P02 was an older adult woman with chronic sleep difficulties characterized by problems with sleep initiation, nocturnal awakenings, and early morning waking, in the context of midlife hormonal changes and emotional distress. During baseline, BSRSQA scores were highly variable and relatively low (median = 18.0). A marked upward shift occurred early in the intervention despite non-individualized parameter settings, with continued variability but a higher median during the non-optimal phase (median = 25.0). Following transition to an individualized optimal ISF-NF parameter setting, scores became more stable and consistently high, indicating a sustained level increase (median = 27.0; Figure D3a). Visual analysis suggested an early response followed by consolidation into a stable high-level improvement.

Participant P03. P03 was a middle-aged woman with persistent sleep disturbances following burnout related to long-term work stress, accompanied by fatigue and cognitive difficulties. During baseline, BSRSQA scores showed moderate variability without a clear trend (median = 20.0). Throughout the intervention, clinicians were unable to establish an individualized optimal ISF-NF parameter setting, and scores remained stable at a similar level during the non-optimal phase (median = 20.0; Figure D3b). Visual analysis indicated a flat trajectory with no meaningful change in level or variability. The absence of an identifiable individualized optimal parameter setting, together with the lack of phase-dependent change, suggests no evident sleep-specific treatment response in this case.

Participant P04. P04 was a middle-aged woman with severe and long-standing sleep difficulties following a major personal crisis, in the context of substantial ongoing external stress. During baseline, BSRSQA scores were high and highly variable, indicating unstable perceived sleep quality (median = 15.0). Only a small number of data points were obtained during the non-optimal intervention phase, and no data were available from a phase with an individualized parameter setting (Figure D3c). Visual analysis was therefore limited, and no conclusions regarding phase-dependent treatment effects could be drawn. Disrupted participation and incomplete data precluded meaningful SCED interpretation in this case.

Figure D3. Daily BSRSQA scores for Participants P02, P03 and P04 across baseline and intervention phases. Dotted vertical lines indicate ISF-NF training days and the corresponding temporal integration (frequency) parameter used in the feedback algorithm. For P03, no individualized optimal

parameter setting was established and scores remained stable across phases. For P04, disrupted participation and incomplete data precluded meaningful visual single-case analysis.

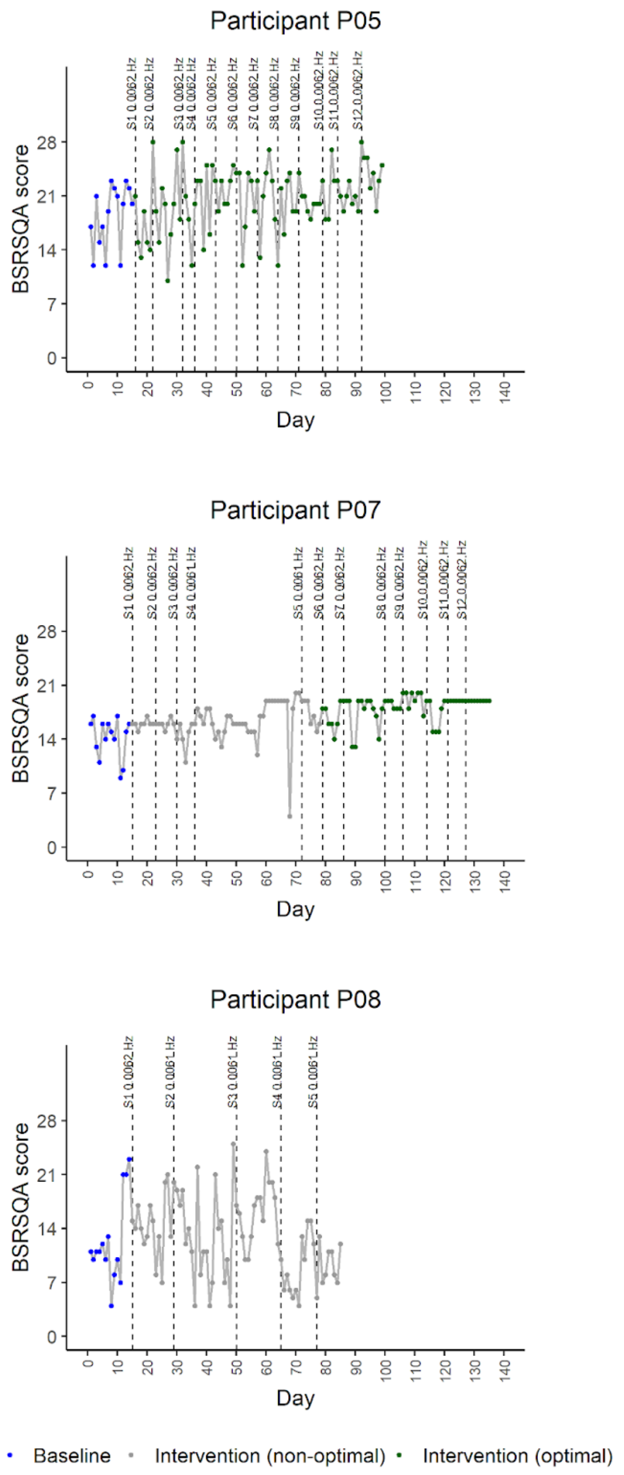

Participant P05. P05 was an elderly man with long-standing sleep difficulties following work-related stress and burnout, accompanied by residual anxiety and cognitive complaints. During baseline, BSRSQA scores were elevated and variable, indicating unstable perceived sleep quality (median = 20.0). From the first session, the participant appeared to be training at an individualized optimal ISF-NF parameter setting, and no non-optimal phase was present (Figure D3d). Over the course of the intervention, visual analysis revealed a slow but consistent upward trend, with higher scores and reduced variability toward the later sessions (median = 21.0).

Participant P07. P07 was a younger middle-aged man with severe and persistent sleep disturbances characterized by frequent nocturnal awakenings related to nightmares. During baseline, BSRSQA scores were moderately elevated and relatively stable (median = 15.0). Scores remained at a similar level during the initial non-optimal intervention phase (median = 16.0). Following transition to the individualized optimal ISF-NF parameter setting, a gradual level increase emerged, with higher and more stable scores toward the later intervention phase (median = 19.0; Figure D3e). Visual analysis indicated a modest phase-dependent improvement. The delayed and gradual nature of change suggests a late-emerging treatment response rather than an immediate effect.

Participant P08. P08 was a young adult man with severe and irregular sleep–wake patterns, characterized by recurrent nights without sleep and pronounced daytime fatigue. During baseline, BSRSQA scores were relatively stable but elevated (median = 11.0). The non-optimal intervention phase was marked by extreme variability (range = 4–24; median = 13.0; Figure D3f). The participant discontinued the study after six sessions, and no data were obtained from a phase with an individualized optimal parameter setting. Visual analysis was therefore limited. In addition, the participant exhibited pronounced sweating during several sessions, which compromised EEG signal stability and likely affected the quality of the neurofeedback training signal. Together with irregular circadian patterns and inconsistent session attendance, these factors resulted in limited treatment exposure and precluded meaningful visual SCED interpretation in this case.

Figure D3f. Daily BSRSQA scores for Participants P05, P07 and P08 across baseline and intervention phases. Dotted vertical lines indicate ISF-NF training days and the corresponding training parameter. Premature discontinuation and irregular participation resulted in limited treatment exposure and no data from an optimal phase.

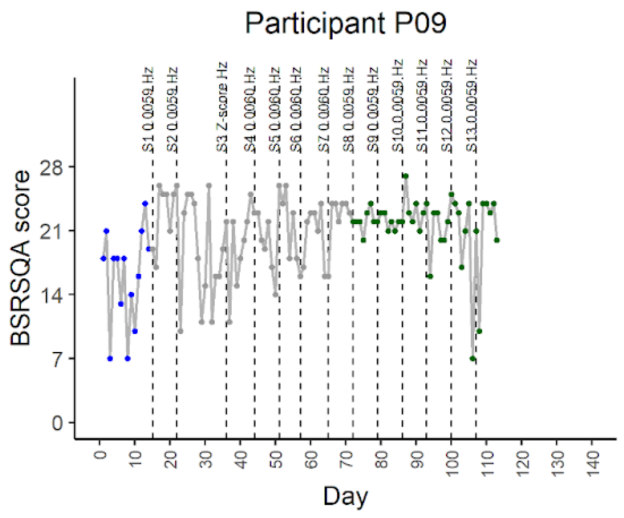

Participant P09. P09 was a middle-aged woman with long-standing stress-related exhaustion and chronic sleep difficulties. During baseline, BSRSQA scores showed pronounced variability, indicating highly unstable perceived sleep quality (median = 18.0). During the initial non-optimal intervention phase, a gradual upward shift emerged alongside reduced variability (median = 22.0). This pattern became more apparent during the phase with an individualized optimal ISF-NF parameter setting, with scores clustering at a higher level and fewer low outliers despite an unchanged median (median = 22.0; Figure D3g). Visual analysis indicated gradual stabilization and improvement over time. The case illustrates how changes in variability and distribution, rather than median values alone, may reflect meaningful treatment-related change.

Figure D3g. Daily BSRSQA scores for Participant P09 across baseline and intervention phases. Dotted vertical lines indicate ISF-NF training days and the corresponding temporal integration (frequency) parameter.

Appendix D3 Visual Interpretation of BSRSQA Trajectories Across Participants

Participant	Baseline pattern	Change during intervention	Post-optimization pattern	Visual interpretation
P01	Moderate, stable	Increased variability	Sustained high-level scores	Clear phase-dependent improvement
P02	Highly variable	Early upward shift	Stable high-level sleep	Early response with sustained improvement
P03	Moderate variability	No change	No optimal phase	Stable trajectory, no clear treatment effect
P04	Highly variable, sparse	Insufficient data	No optimal phase	Data insufficient for interpretation

P05	Elevated, fluctuating	Gradual increase	Reduced variability late	Gradual gains without phase shift
P06	Fluctuating baseline	Early improvement	Stable high-level sleep	Sustained improvement post-optimization
P07	Moderately elevated, stable	Minimal change	Gradual late-phase improvement	Modest improvement emerging over time
P08	Relatively stable	Extreme variability	Dropout	Marked instability, limited exposure
P09	Highly variable	Gradual improvement	Stabilization at higher level	Gradual stabilization despite stressors
P10	Highly variable	No clear level change	Reduced variability	Increased stability without quality shift

E. Materials and Surveys

Appendix E1. Symptom Monitoring Checklist: 24- and 48-Hour Post-Training Feedback

The following checklist was used to monitor subjective symptoms and autonomic reactions within 24 and 48 hours after each neurofeedback session. Participants rated symptom intensity on a three-point scale.

Symptom	1 (Mild)	2 (Moderate)	3 (Severe)
Emotional reactivity (e.g., irritability, aggression)	☐	☐	☐
Emotional sensitivity (e.g., sadness, tearfulness)	☐	☐	☐
Difficulty falling asleep	☐	☐	☐
Lack of deep sleep	☐	☐	☐
Agitated, speeded up	☐	☐	☐
Sedated, slowed down	☐	☐	☐
Physical tension or muscle spasms	☐	☐	☐

Dizziness	☐	☐	☐
Nightmares or night terrors	☐	☐	☐
Sugar craving	☐	☐	☐
Reduced appetite (eating less than usual)	☐	☐	☐
Headache	☐	☐	☐
Nausea	☐	☐	☐
Diarrhea (other than usual)	☐	☐	☐
Constipation (other than usual)	☐	☐	☐
Other (please specify): __________	☐	☐	☐

Note. This checklist was used as a clinical monitoring tool to guide individualized adjustment of the parameter setting and to identify potential non-optimal responses. Checklist data were used descriptively and were not subjected to statistical analysis.

Appendix E2. Subjective Sleep Rating (BSRSQA)

Please think back on how you slept last night and indicate your experience by marking the scales below. Ratings toward the left indicate lower satisfaction, and ratings toward the right indicate higher satisfaction.

**Sleep dimension**	**1**	**2**	**3**	**4**	**5**	**6**	**7**
Sleep onset (ease of falling asleep)	☐	☐	☐	☐	☐	☐	☐
Sleeping through the night without awakenings	☐	☐	☐	☐	☐	☐	☐
Feeling restored by sleep	☐	☐	☐	☐	☐	☐	☐
Overall experience of the night’s sleep	☐	☐	☐	☐	☐	☐	☐

Scale anchors:
1 = Very low satisfaction
7 = Very high satisfaction

Note. This daily subjective sleep rating was used as the primary outcome measure in the single-case analyses to capture perceived sleep quality across multiple dimensions.

F. Methodological Transparency

Appendix F1. Alignment With the CRED-nf Checklist (Ros et al., 2020)

This appendix provides an overview of how relevant domains from the CRED-nf checklist (Ros et al., 2020) are addressed in the present single-case experimental design (SCED) study. The checklist was used as a guiding framework to enhance transparency and methodological clarity in the design, conduct, and reporting of the study. As some checklist items were originally developed for group-based neurofeedback studies, certain domains are only partially applicable to the present SCED design.

This report was generated using the CRED-nf online checklist (version 1.0), adapted from the original CRED-nf manuscript available at https://doi.org/10.1093/brain/awaa009.

CRED-nf checklist summary

14 March, 2026

Manuscript title: Infra-Slow EEG Neurofeedback for Insomnia: A Single-Case Experimental Study in Primary Care
Corresponding Author: Arne Edvardsson
Corresponding author email: arne.edvardsson@vgregion.se

Item No.	Checklist item	Manuscript Details
**Pre-experiment**		
1a	Pre-register experimental protocol and planned analyses	Prospectively registered at Researchweb (VGR project ID 282982)
1b	Justify sample size	Ten participants were recruited from the routine patient flow at a primary care center in Västra Götaland, Sweden, during 2024. In SCED research, evidence is typically established through systematic replication across a relatively small number of participants rather than through large sample sizes (Tate et al., 2017). The

		number of participants in the present study was therefore considered sufficient to allow replication of intervention effects across multiple individual time series.

Control groups

2a	Employ control group(s) or control condition(s)	The present study was based on a single-case experimental design (SCED) with repeated measures (Krasny-Pacini and Evans, 2018). The study followed an AB design. The baseline phase (A) lasted at least 14 days and included daily sleep measurements. The intervention phase (B) started with all participants receiving the ISF-NF intervention, beginning with basic sleep hygiene information.
2b	When leveraging experimental designs where a double-blind is possible, use a double-blind	NA: A double-blind was not appropriate for this experiment
2c	Blind those who rate the outcomes	Those who rated the outcome were not blind to group assignment
	Blind those who analyse the data	Those who analysed the data were not blind to group assignment
2d	Examine to what extent participants and experimenters remain blinded	No measures were taken to examine whether participants and experimenters remained blind
2e	In clinical efficacy studies, employ a standard-of-care intervention group as a benchmark for improvement	NA: This is not a clinical efficacy study

3a	Collect data on psychosocial factors	Participants were asked to complete questions about housing, age, economic status, and medical history using a Demographic Questionnaire. M.I.N.I. is a structured interview specifically developed to screen for DSM-IV and ICD-10 psychiatric diagnoses (Sheehan et al., 1998). Current versions are aligned with DSM-5 and retain an administration time of about 15 minutes (Lecrubier et al., 1997). Daily subjective sleep quality was assessed with the BSRSQA, a brief four-item measure (1–7 per item) developed by the first and last authors of the present manuscript for this study. As a study-developed instrument, it has no prior citation; full item wording and scoring are provided in Appendix E2. The PSQI allows participants to evaluate their sleep quality over a one-month period (Buysse et al., 1989). It consists of 19 questions that can be divided into seven components. Buysse et al. (1989) reported that the PSQI had high sensitivity and specificity in distinguishing between good and poor sleepers. The therapeutic alliance was measured after each intervention session using the WAI-SR (Munder et al., 2010). Responses are rated on a scale from 1 (not at all) to 7 (completely). The PHQ-9 is an instrument used to screen for and assess the severity of depression based on DSM-IV criteria (Sun et al., 2020). Responses range from 0 to 3 (0 = not at all; 1 = several days; 2 = more than half the days; 3 = nearly every day). Participants are categorized into four levels of depression: 5–9 (mild), 10–14 (moderate), 15–19 (moderately severe), and 20+ (severe). Previous studies have shown a sensitivity of approximately 90% and a specificity of around 77%–88% (Hansson et al., 2009). The GAD-7 measures symptoms of anxiety (Spitzer et al., 2006). It includes seven standard questions and an optional eighth question about functional impairment. Participants respond to questions such as "Have you been feeling nervous, anxious, or on edge?" and "Have you had trouble relaxing?" on a scale from 0 (not at all) to 3 (nearly every day). The optional question asks, "If these symptoms occurred, how much did they affect your ability to work, carry out home tasks, and engage in relationships?" This is rated on a four-point scale from "not at all" to "extremely difficult." The questionnaire has demonstrated strong psychometric properties (Johnson et al., 2019). The CSQ-8 includes eight questions about patient satisfaction (Attkisson and Zwick, 1982). Responses range from 1 to 4, with 4 being the highest level of satisfaction. The questionnaire has shown high

		reliability and validity (Attkisson and Zwick, 1982). The NEQ includes 32 questions about adverse events and effects of psychological treatment (Rozental et al., 2016). Participants rate the impact of negative treatment events on a scale from "not at all" to "extremely." They are also asked whether they believe the negative effects were caused by the treatment.
3b	Report whether participants were provided with a strategy	During ISF-NF training sessions, participants were seated in a reclining chair while electrodes were positioned (three on the scalp: two active and one ground, and one electrode on each ear as reference). Either a headband or Ten20 conductive paste was used to ensure stable fixation of the electrodes. Participants were encouraged to make themselves comfortable and to inform the therapist if they experienced any discomfort during the session. Based on participant feedback, in-session adjustments could be made by the therapist. Training was conducted with eyes closed. Participants were instructed to simply sit quietly and listen to the auditory feedback signal. They were informed that the signal reflected ongoing brain activity and that they were not expected to actively control or manipulate it. Instead, participants were encouraged to adopt a relaxed and curious attitude while passively observing the feedback.
3c	Report the strategies participants used	The strategies participants used were not recorded or not reported in the manuscript
3d	Report methods used for online-data processing and artifact correction	2.6.1 Equipment and Software The NF amplifier equipment used for EEG screening andISF-NF training was a BrainMaster 2EB+ 2×2 two-channel EEG amplifier operated with BrainAvatar™ software (version 4.7.5; BrainMaster Technologies Inc., Bedford, OH, USA), together with the qEEG-Pro database developed in the Netherlands. NF training was delivered using auditory feedback generated through algorithmic processing of the DC-coupled bipolar EEG signal. The feedback was based on temporal integration of slow signal dynamics rather than direct representation of a specific spectral oscillation. Signal quality was monitored throughout the sessions, and electrode placement or impedance was adjusted when necessary to minimize recording artefacts.
3e	Report condition and group effects for artifacts	Condition and group effects for artifacts were not measured, or not reported in the manuscript

4a	Report how the online-feature extraction was defined	All participants began training using an initial temporal integration parameter corresponding to approximately 0.0062 Hz (or 0.0059 Hz in cases with a history of migraine). This parameter represents a temporal integration setting within the feedback algorithm, defining the time scale over which slow fluctuations in the EEG signal are integrated for feedback generation. During the course of training, the parameter was adjusted in small increments of 0.0001 Hz based on participants' in-session responses and analyzed self-reports completed between sessions.<br><br>The feedback algorithm integrates slow fluctuations in a DC-coupled bipolar EEG signal over a defined time scale. The feedback signal was derived by comparing a rapidly updating estimate of the bipolar EEG signal with a slower exponentially damped moving average, allowing slow amplitude transitions to be reflected in auditory feedback.
4b	Report and justify the reinforcement schedule	Neurofeedback training was delivered using continuous auditory feedback derived from slow fluctuations in a DC-coupled bipolar EEG signal. Participants listened to two tones representing the direction of the slow signal dynamics. The reinforcement schedule was therefore continuous. The temporal integration parameter defining the time scale of the feedback signal was adjusted in small increments (0.0001 Hz) across sessions based on participants' responses and self-reported effects.
4c	Report the feedback modality and content	The feedback signal was derived by comparing a rapidly updating estimate of the bipolar EEG signal with a slower exponentially damped moving average, allowing slow amplitude transitions to be reflected in auditory feedback. Participants listened to two different tones representing the direction of these slow fluctuations in the feedback signal derived from the bipolar EEG recording across the two active electrode sites (Balt et al., 2020; Bekker et al., 2021).
4d	Collect and report all brain activity variable(s) and/or contrasts used for feedback, as displayed to experimental participants	The feedback signal was derived by comparing a rapidly updating estimate of the bipolar EEG signal with a slower exponentially damped moving average, allowing slow amplitude transitions to be reflected in auditory feedback. Participants listened to two different tones representing the direction of these slow fluctuations in the feedback signal derived from the bipolar EEG recording across the

		two active electrode sites (Balt et al., 2020; Bekker et al., 2021).
4e	Report the hardware and software used	2.6.1 Equipment and Software The NF amplifier equipment used for EEG screening and ISF-NF training was a BrainMaster 2EB+ 2×2 two-channel EEG amplifier operated with BrainAvatar™ software (version 4.7.5; BrainMaster Technologies Inc., Bedford, OH, USA), together with the qEEG-Pro database developed in the Netherlands. NF training was delivered using auditory feedback generated through algorithmic processing of the DC-coupled bipolar EEG signal. The feedback was based on temporal integration of slow signal dynamics rather than direct representation of a specific spectral oscillation. Signal quality was monitored throughout the sessions, and electrode placement or impedance was adjusted when necessary to minimize recording artefacts.

Outcome measures - brain

5a	Report neurofeedback regulation success based on the feedback signal	The manuscript does not report neurofeedback regulation success based on the feedback signal
5b	Plot within-session and between-session regulation blocks of feedback variable(s), as<br><br>well as pre-to-post resting baselines or<br><br>contrasts	The manuscript does not plot within-session and between-session regulation blocks of feedback variable(s), as well as pre-to-post resting baselines or contrasts
5c	Statistically compare the experimental condition/group to the control<br><br>condition(s)/group(s) (not only each group to baseline measures)	The manuscript does not statistically compare the experimental condition/group to the control condition(s)/group(s)

6a	Include measures of clinical or behavioural significance, defined a priori, and describe whether they were reached	4.7 Conclusions This exploratory SCED study conducted in primary care suggests that ISF-NF may be associated with meaningful improvements in perceived sleep quality for a subset of individuals with insomnia. Within-person analyses revealed heterogeneous yet interpretable change trajectories, highlighting the relevance of individualized parameter adjustment in adaptive ISF-NF protocols. Objective wearable-derived data did not uniformly align with changes in total sleep time but indicated that shifts in sleep organization, including improved consolidation and timing, may represent important dimensions of improvement. These findings reinforce the multidimensional nature of sleep health and the importance of integrating subjective and objective measures when evaluating treatment response.
6b	Run correlational analyses between regulation success and behavioural outcomes	3.3 SCED Results: Individual Sleep Trajectories 3.3.1 Overview of Individual Trajectories in Subjective Sleep Quality (BSRSQA) In general the individual graphs for BSRSQA indicate that most participants improved their perceived sleep (see Figure 2).<br><br>3.4.2 Sleep Consolidation and Fragmentation Visual inspection of actogram-style sleep timing plots revealed marked inter-individual differences in baseline sleep organization. Among participants who initially exhibited fragmented sleep characterized by multiple discrete nocturnal segments, a descriptive reduction in the number of separate sleep episodes was observed over the course of the intervention (see Figure 4), suggesting increased sleep consolidation in a subset of cases. Figure 4. Sleep consolidation and timing plots across baseline and intervention phases.

7a	Upload all materials, analysis scripts, code	No additional materials, analysis scripts, code, or raw data are publicly available for this manuscript.

Data storage

7a	Upload all materials, analysis scripts, code	Data are not publicly available but may be made available from the corresponding author upon reasonable request.

Appendix F2. Ethical Approval

Ethical approval for the study was granted by the Swedish Ethical Review Authority (Etikprövningsmyndigheten), approval number 2023-05411-01. The study was conducted in accordance with the Declaration of Helsinki.
